# Symbiotic bracovirus of a parasite manipulates host lipid metabolism via tachykinin signaling

**DOI:** 10.1371/journal.ppat.1009365

**Published:** 2021-03-01

**Authors:** Yanping Wang, Xiaotong Wu, Zehua Wang, Ting Chen, Sicong Zhou, Jiani Chen, Lan Pang, Xiqian Ye, Min Shi, Jianhua Huang, Xuexin Chen

**Affiliations:** 1 Institute of Insect Sciences, College of Agriculture and Biotechnology, Zhejiang University, Hangzhou, China; 2 Ministry of Agriculture Key Lab of Molecular Biology of Crop Pathogens and Insect Pests, Zhejiang University, Hangzhou, China; 3 Key Laboratory of Biology of Crop Pathogens and Insects of Zhejiang Province, Zhejiang University, Hangzhou, China; 4 State Key Lab of Rice Biology, Zhejiang University, Hangzhou, China; Wageningen University, NETHERLANDS

## Abstract

Parasites alter host energy homeostasis for their own development, but the mechanisms underlying this phenomenon remain largely unknown. Here, we show that *Cotesia vestalis*, an endoparasitic wasp of *Plutella xylostella* larvae, stimulates a reduction of host lipid levels. This process requires excess secretion of *P*. *xylostella* tachykinin (PxTK) peptides from enteroendocrine cells (EEs) in the midgut of the parasitized host larvae. We found that parasitization upregulates PxTK signaling to suppress lipogenesis in midgut enterocytes (ECs) in a non-cell-autonomous manner, and the reduced host lipid level benefits the development of wasp offspring and their subsequent parasitic ability. We further found that a *C*. *vestalis* bracovirus (CvBV) gene, *CvBV 9–2*, is responsible for *PxTK* induction, which in turn reduces the systemic lipid level of the host. Taken together, these findings illustrate a novel mechanism for parasite manipulation of host energy homeostasis by a symbiotic bracovirus gene to promote the development and increase the parasitic efficiency of an agriculturally important wasp species.

## Author summary

Parasitic wasps are ubiquitous on earth and diverse. They lay eggs in or on the bodies of their hosts, and they have evolved adaptive strategies to regulate the energy metabolism of their hosts to match their own specific nutrition requirements. Here, we found that *Cotesia vestalis*, a solitary endoparasitoid of *Plutella xylostella*, uses symbiotic bracovirus as a weapon to manipulate host systemic lipid levels. Specifically, a *C*. *vestalis* bracovirus (CvBV) gene, *CvBV 9–2*, is responsible for the induction of PxTK, which in turn suppresses lipogenesis in the midgut of the parasitized host, leading to a nutritional lipid level suitable for the development and subsequent parasitic efficiency of *C*. *vestalis* wasps. Our study provides innovative insights into the mechanisms by which parasitic wasps manipulate host lipid homeostasis and may help to expand our knowledge of other parasitic systems.

## Introduction

Parasitism is common in nature, and all living organisms are vulnerable to parasites [1]. Parasites depend on their hosts, which provide them with nutrition and habitat, for their development [2–6]. Thousands or even millions of years of coevolution have driven parasites to display very complex and exquisite strategies to actively manipulate host metabolism to make the host more suitable to sustaining their specific nutritional requirements [7–11]. Although alteration of host energy homeostasis by parasites is a widespread phenomenon, the underlying mechanisms remain largely unknown.

Parasitic insects, particularly parasitic wasps (also known as parasitoids), lay eggs on or inside the body of the host, and hatched wasp larvae acquire their nutrients directly from the host during all of their immature development, which eventually results in the death of the host [11–13]. As such, many parasitoids are used as biological control agents for insect pests. Previous studies have revealed that parasitoids produce various factors, including venom, polydnaviruses (PDVs) and teratocytes, that benefit the survival of wasp offspring by disabling host cellular and humoral immune defenses [14–18]. Parasitic factors have also been reported to modify host energy metabolism, which in turn provides the parasite with needed nutrients and leads to successful parasitism [19–23].

PDVs are a special type of large double-stranded DNA virus in parasitic insects that do not replicate in infected hosts [15,18,24–27]. They are mainly classified into two different genera: bracovirus (BV), a widespread group of braconid parasitoid symbiotic viruses, and ichnovirus (IV), a widespread group of ichneumonid parasitoid symbiotic viruses [18,24,27–30]. The life cycle of PDVs is generally divided into two parts: one is in their primary host (the wasp), in which the viral DNAs are dramatically replicated only in the nuclei of ovarian calyx cells of female wasps, and the other is in their secondary host (usually a caterpillar), in which the viruses integrate into host genome and subsequently express the numerous virulence genes to regulate the host physiology [26,27,31]. To date, the genomes of nine BV species and five IV species have been fully sequenced [25,32–41]. These studies provided comprehensive information on PDV genes and have accelerated functional investigations of individual virus genes. Recently, it was reported that two PDV genes, *VHv1*.*1* and *VHv1*.*4*, members of the Cys-motif gene family of *Campoletis sonorensis* ichnovirus (CsIV), might affect host cellular immune responses [42], and another PDV gene, *TnBVANK1*, a member of the viral ankyrin gene family of *Toxoneuron nigriceps* bracovirus (TnBV), might inhibit host humoral immune responses [43,44]. In addition, some PDV genes have been found to regulate the development of their hosts. For instance, *TnBVANK3* is responsible for the arrest of the host larval-pupal transition by impairing the expression of ecdysone biosynthesis genes [45]. However, few studies have focused on the crucial roles of PDV genes in manipulating host energy metabolism.

The intestine is a key endocrine organ and central regulator of nutrient uptake and energy homeostasis. Intestinal lipid metabolism is critical for fine-tuning systemic lipid levels in the larval stage of holometabolous insects [46–49]. Recent studies have shown that enteroendocrine cells (EEs) can secrete multiple gut hormones in response to the nutritional status of the organism and orchestrate systemic metabolism [49–51]. Tachykinins (TKs) are the most abundant secreted peptides expressed in midgut EEs. The induction of TKs in EE cells inhibits intestinal lipid production and subsequently reduces systemic lipid levels by repressing lipogenesis in intestinal enterocytes (ECs), in association with the TKR99D receptor and protein kinase A (PKA) signaling [49].

*Cotesia vestalis* (Hymenoptera: Braconidae) is a solitary endoparasitoid of *Plutella xylostella*, which is a worldwide migratory pest that attacks nearly all cruciferous vegetable crops and causes severe economic losses [52–54]. Here, we report a crucial role of a *C*. *vestalis* polydnavirus, named *C*. *vestalis* bracovirus (CvBV), which reduces host systemic lipid levels. We further found that one CvBV gene, *CvBV 9–2*, suppressed host lipogenesis by upregulating TK levels within the midgut of the parasitized host larvae. Failure to reduce host lipid levels during parasitism resulted in delayed development of *C*. *vestalis* larvae and reductions in the female ratio of wasp offspring and the subsequent ability of the female offspring to parasitize.

## Results

### *Cotesia vestalis* parasitization promotes the reduction of host systemic lipid levels

**Fig 1:**
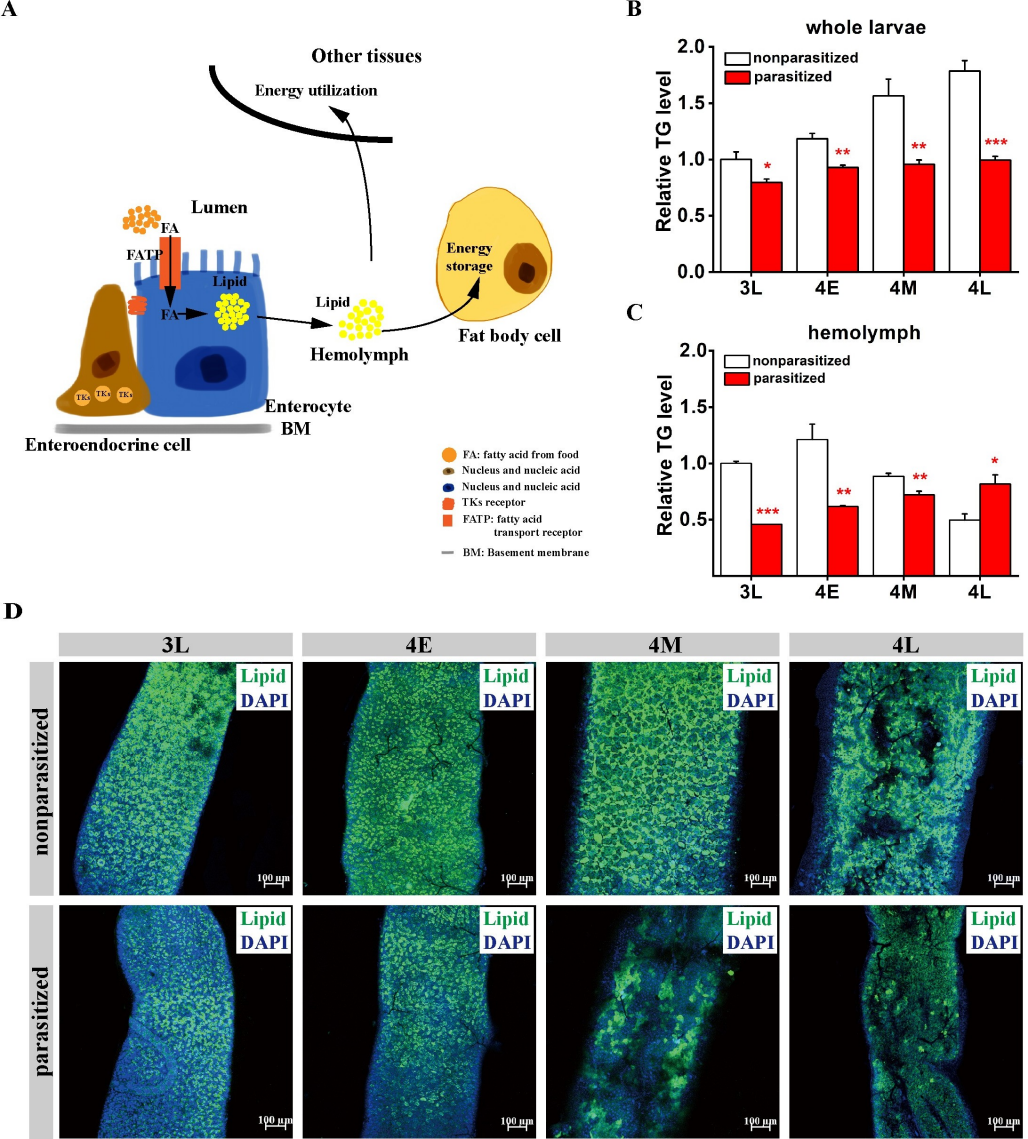
Parasitization by *Cotesia vestalis* influences systematic lipid contents and intestinal lipid droplet accumulation in *Plutella xylostella* larvae. (A) Schematic diagram of the procedures for lipid synthesis, transportation, storage and utilization, modified from Kamareddine et al [88]. Tachykinins (TKs) in midgut enteroendocrine cells (EEs) could suppress lipogenesis in intestinal enterocytes (ECs) via TK receptors. (B) Levels of triglycerides (TGs) in *C*. *vestalis*-parasitized and nonparasitized host larvae among different developmental stages (n = 10 for each group). Data were analyzed by Tukey’s test. Values represent the means ± SD of three independent experiments (*: p < 0.05; **: p < 0.01; ***: p < 0.001). 3L: Late 3^rd^ instar; 4E: Early 4^th^ instar; 4M: Middle 4^th^ instar; 4L: Late 4^th^ instar. (C) Relative levels of TG in hemolymph from *C*. *vestalis*-parasitized and nonparasitized host larvae among different developmental stages (n = 30 for each group). Data were analyzed by Tukey’s test. Values represent the means ± SD of three independent experiments (*: p < 0.05; **: p < 0.01; ***: p < 0.001). (D) Fluorescent images of the middle regions of midguts from 3L, 4E, 4M and 4L *C*. *vestalis*-parasitized and nonparasitized *P*. *xylostella* larvae. Lipids were stained with BODIPY (green), and nuclei were labeled with DAPI (blue). Scale bars: 100μm.

In insects, fatty acids taken in from the diet are absorbed by ECs and resynthesized into lipids and packaged into lipoprotein particles that are transported to fat bodies for storage and other tissues for direct energy supply [55–58] (Fig 1A). Parasites have been extensively found to evolve to increase or reduce the systemic lipid levels of their hosts to facilitate their own infection, proliferation, development and reproduction [20,59]. To test whether *C*. *vestalis* parasitization affects host *P*. *xylostella* lipid metabolism, we measured the whole-body triglyceride (TG) levels in *P*. *xylostella* at different immature stages, including the 3^rd^ instar late stage (3L), 4^th^ instar early stage (4E), 4^th^ instar middle stage (4M) and 4^th^ instar late stage (4L). Compared with the nonparasitized larvae, the parasitized *P*. *xylostella* host larvae showed a significant reduction in whole-body TG levels at all four stages, with an almost 50% reduction in the 4L stage (Fig 1B). We next evaluated the changes in circulating TG in the host hemolymph post *C*. *vestalis* infection and found that the levels were also dramatically decreased in the parasitized *P*. *xylostella* larvae at the 3L, 4E and 4M stages (Fig 1C). Interestingly, there was an increase in circulating TG at the 4L stage compared to that in the nonparasitized larvae. In caterpillars, most TGs are stored in the fat body and are essential for surviving periods of starvation and conditions that preclude the uptake of nutrients, such as metamorphosis [60]. Thus, one possible explanation for this observation could be that the fat bodies of the parasitized host larvae were dissolved to release the lipids into the hemolymph at the late fourth stage, indicating that *C*. *vestalis* wasp larvae require large amounts of lipid resources for their development at this specific stage.

To ascertain whether the decrease in lipid content was a consequence of a decrease in lipid production in the host intestine, we used the neutral lipid dyes Oil Red O and BODIPY to stain the midgut of *P*. *xylostella*. The results from both Oil Red O and BODIPY staining showed that neutral lipid droplets (LDs) were most abundant in the ECs located in the middle and posterior regions of the midgut (Figs 1D and S1). Strikingly, we observed a dramatic decrease in the amount of lipid staining in midgut ECs in all four stages after *C*. *vestalis* parasitism compared to that of CK. Taken together, our observations indicate that *C*. *vestalis* parasitization decreases intestinal lipid production, which results in the reduction of host systemic lipid levels.

### PxTK signaling regulates lipid metabolism in the midgut of *P*. *xylostella* larvae

**Fig 2:**
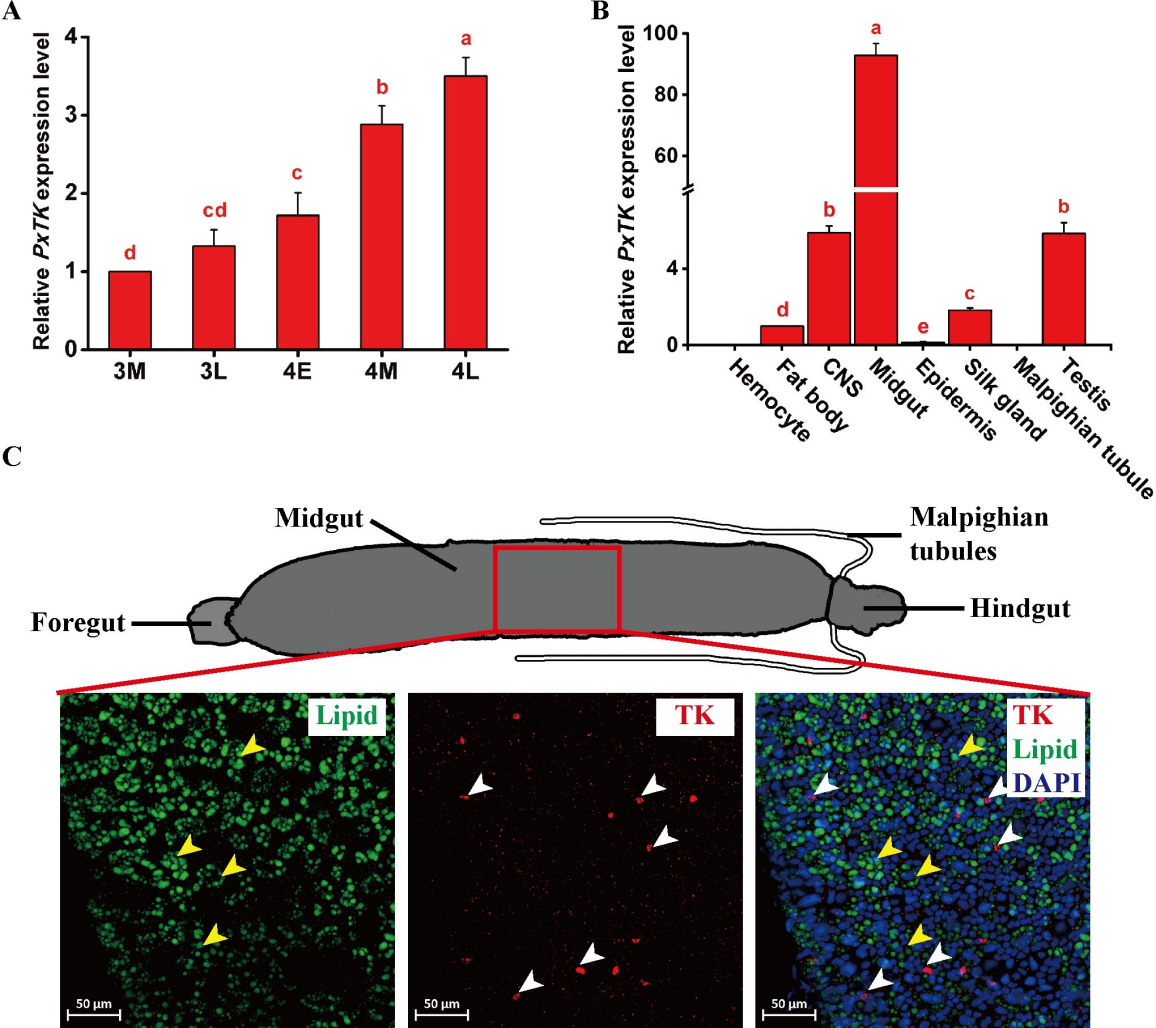
*Plutella xylostella* TK is highly expressed in the midgut. (A) Relative mRNA levels of *PxTK* in *P*. *xylostella* larvae among different developmental stages (n = 5 for each group). Values represent the means ± SD of three independent experiments. 3M: Middle 3^rd^ instar; 3L: Late 3^rd^ instar; 4E: Early 4^th^ instar; 4M: Middle 4^th^ instar; 4L: Late 4^th^ instar. (B) Relative mRNA levels of *PxTK* in eight different tissues of 4L *P*. *xylostella* larvae (n = 30 per tissue). Values represent the means ± SD of three independent experiments. (C) Upper panel: Schematic diagram of the *P*. *xylostella* larval gut; Lower panel: Localization of TK in the midgut (anti-TK, red). TK was localized in the cytoplasm in EEs (white arrowheads). ECs were labeled by BODIPY (green, yellow arrowheads), and nuclei were labeled by DAPI (blue). Scale bars: 50μm.

We wondered whether lipid metabolism in *P*. *xylostella* is regulated by TKs, as described in *Drosophila* [49]. To address this question, we cloned the full-length cDNA of the *P*. *xylostella* tachykinin (*PxTK*) precursor gene, which has three exons and a total length of 1044 bp (S2A Fig). The predicted PxTK protein has 296 amino acids, including a 29 amino acid signal peptide at the N-terminus (S2B Fig). Six putative *P*. *xylostella* TK peptides (PxTKs) were defined by the common flanking dibasic cleavage sites (combinations of K and R). In addition, a conserved C-terminal motif was identified from individual mature peptides with the sequence FxGxR (x is a variable residue) (S3 Fig). To assess the function of PxTKs in *P*. *xylostella* larvae, we first characterized the expression profiles of *PxTK* in the different stages from 3M to 4L. The results show that the level of *PxTK* gradually increases during *P*. *xylostella* development (Fig 2A). We further quantified the levels of *PxTK* in different tissues from 4L *P*. *xylostella* larvae and found that *PxTK* had the highest expression in the midgut, while the expression of *PxTK* was also higher in the CNS and testis than in the other tissues (Fig 2B). The results were consistent with previous findings that *TK* is a brain-gut hormone gene [61,62]. The results of immunofluorescence assays show that PxTK is produced by EEs in the midgut but not by ECs in *P*. *xylostella* host larvae (Fig 2C). In particular, more EEs in the middle region of the larval midgut were found to secrete PxTKs than in the anterior and posterior sides (S4 Fig).

**Fig 3:**
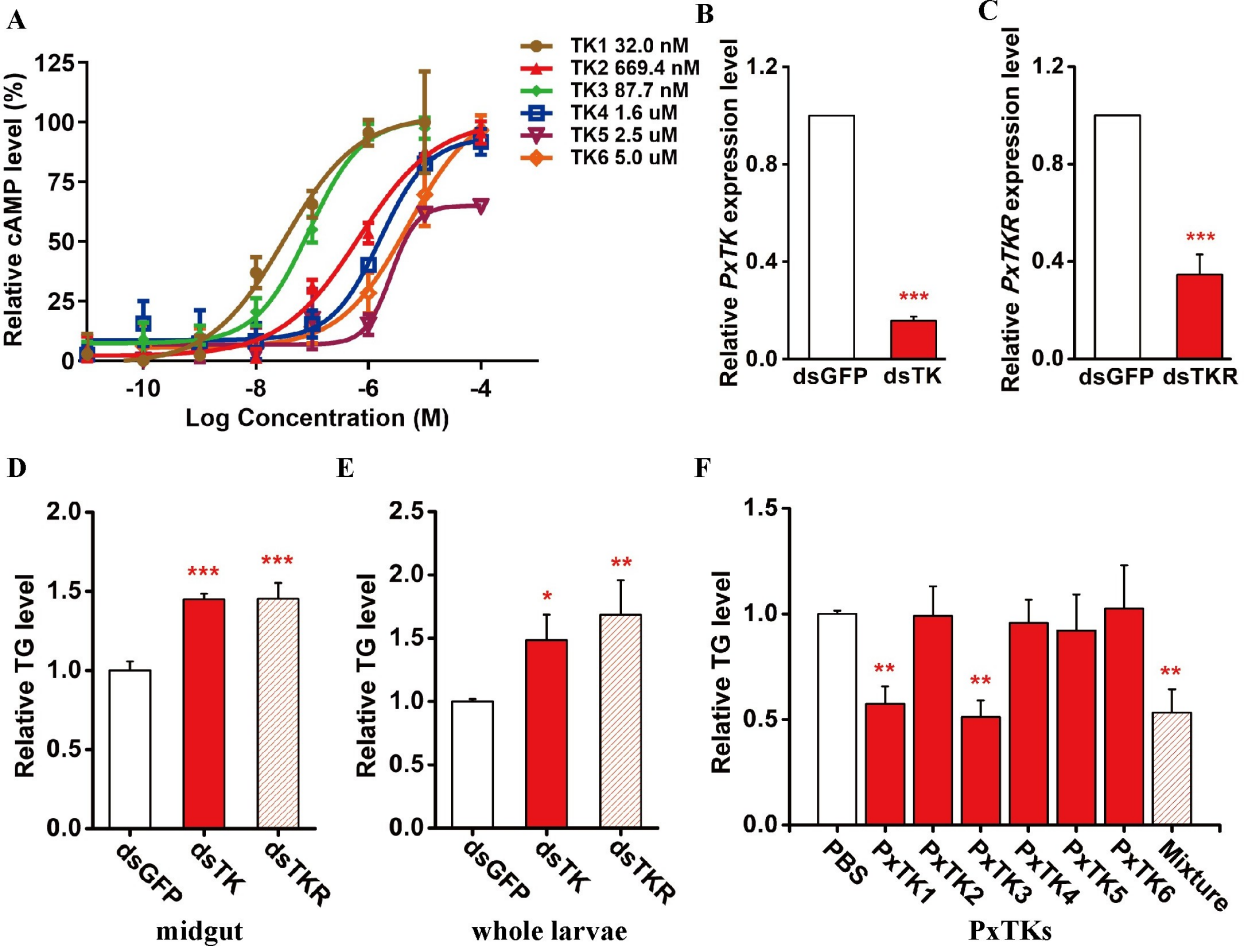
*PxTK/PxTKR* signaling plays critical roles in maintaining lipid metabolism. (A) Dose-response curves of HEK293 cells stably expressing PxTKR and treated with PxTKs. All data were taken from at least three independent experiments. Values represent the means ± SD of three independent experiments. The 50% effective concentration (EC_50_) for each PxTK peptide is shown on the right. (B) Relative mRNA levels of *TK* in *P*. *xylostella* larvae at 3 days post *PxTK* silencing with dsGFP treatment as a control (n = 5 for each group). Three biological replicates were performed. Data are the means ± SD; significance was determined by Student’s t-test (***: p < 0.001). (C) Relative mRNA levels of *TKR* in *P*. *xylostella* larvae at 3 days post *PxTKR* silencing with dsGFP treatment as a control (n = 5 for each group). Three biological replicates were performed. Data are the means ± SD; significance was determined by Student’s t-test (***: p < 0.001). (D) Relative levels of triglycerides (TGs) in midguts from dsPxTK-, dsPxTKR- and dsGFP (CK)-treated *P*. *xylostella* larvae at 3 days post microinjection (n = 30 for each group). Data were analyzed by Tukey’s test. Values represent the means ± SD of five independent experiments (***: p < 0.001). (E) Relative levels of TG from the whole body of dsPxTK-, dsPxTKR- and dsGFP (CK)-treated *P*. *xylostella* larvae at 3 days post microinjection (n = 10 for each group). Data were analyzed by Tukey’s test. Values represent the means ± SD of three independent experiments (*: p < 0.05). (F) Relative levels of TG from the whole body of *P*. *xylostella* larvae at 3 days post microinjection of chemically synthesized individual PxTK peptides (TK1, TK2, TK3, TK4, TK5 and TK6) and their mixture (n = 5 for each group). Data were analyzed by Tukey’s test. Values represent the means ± SD of three independent experiments (**: p < 0.01).

The receptor for the tachykinin peptide has been cloned in many insect species and is activated by TKs to trigger intracellular Ca^2+^ mobilization and secondary cAMP accumulation, which in turn stimulate downstream signaling pathways [63,64]. We next cloned the full-length cDNA of the *P*. *xylostella* tachykinin receptor (*PxTKR*), which is a classical G-protein-coupled receptor gene. Two divergent cell expression systems, the mammalian cell line HEK293 and insect cell line Sf21, have been widely used for functional characterization of TK receptors [63]. Here, we constructed a pcDNA 3.1^(+)^/*PxTKR* plasmid and transfected it into HEK293 cells. To verify the cell localization of PxTKR, an antibody was generated, and the staining results showed that the PxTKR signal colocalized with the cell membrane (S5 Fig). To confirm whether the cloned PxTKR was the functional receptor for PxTKs, a cAMP assay was performed by adding six putative mature TKs with different concentrations ranging from 10 pM to 100 μM. The results showed that all PxTKs can activate PxTKR in HEK293 cells in a concentration-dependent manner (Figs 3A and S6). Specifically, PxTK1 was the most active peptide, with a 50% effective concentration (EC_50_) value of 32.0 nM; the next most highly effective TK was PxTK3, with an EC_50_ value of 87.7 nM. The EC_50_ values of the two PxTKs were much lower than those of the other TK peptides (Fig 3A). These results strongly suggest that PxTKR is the receptor of PxTKs. Then, we performed RNA interference (RNAi) in *P*. *xylostella* larvae to knock down the expression of *PxTK* and *PxTKR*. As shown in Fig 3B and 3C, the mRNA levels of *PxTK* and *PxTKR* were significantly reduced in the knockdown larvae compared to the control (*dsGFP*). Strikingly, we observed a dramatic increase in neutral lipid levels in the midgut and whole body after reducing PxTK signaling activity in the EEs of *P*. *xylostella* larvae (Fig 3D and 3E). To avoid off-target effects, another nonoverlapping dsRNAs (dsPxTK_604-877_) were synthesized for *PxTK*. Similarly, the neutral lipid levels were significantly increased in the dsPxTK_604-877_-treated whole host larvae (S7 Fig). To determine which mature PxTK peptide was responsible for the regulation of lipid metabolism in the midgut of *P*. *xylostella*, six individual synthetic peptides and the mixture were injected into the 3M host larvae. The results show that PxTK1 and PxTK3 could suppress the systemic lipid level, and lipid reduction was also found after injecting the mixture of all six peptides (Fig 3F). Through alignments, we found that the amino acid sequences of PxTK1 and PxTK3 were more similar to each other than to other TKs. There were only two amino acid differences between TK1 and TK3 (S8 Fig). In addition, PxTK2, PxTK 4, PxTK 5 and PxTK6 had different N-terminal motifs, which might influence their affinity for PxTKR. Interestingly, the lipid depressive effects of PxTK3 were concentration dependent, while the depressive effects of PxTK1 were not (S9 Fig). Collectively, our results suggest that two of the PxTKs, TK1 and TK3, regulate lipid metabolism in the midgut through their receptor, PxTKR.

### *C*. *vestalis* parasitization increases *PxTK* levels in host larvae

**Fig 4:**
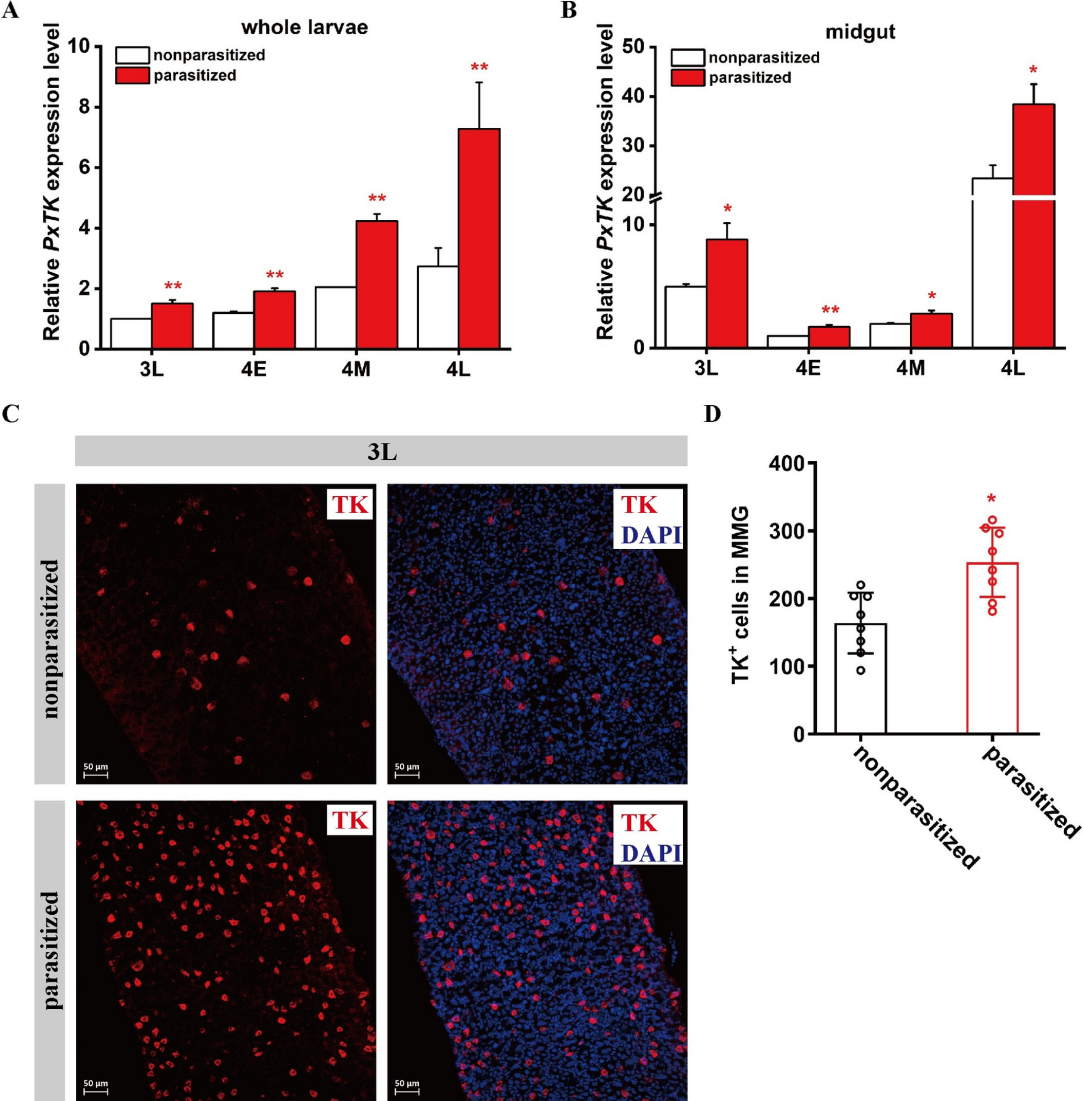
*Cotesia vestalis* parasitization upregulates host intestinal TK expression. (A) Relative mRNA levels of *PxTK* in *C*. *vestalis*-parasitized and nonparasitized host larvae among different developmental stages (n = 5 for each group). Data were analyzed by Tukey’s-test. Values represent the means ± SD of three independent experiments (**: p < 0.01). 3L: Late 3^rd^ instar; 4E: Early 4^th^ instar; 4M: Middle 4^th^ instar; 4L: Late 4^th^ instar. (B) Relative mRNA levels of *PxTK* in midguts from *C*. *vestalis*-parasitized and nonparasitized host larvae at different developmental stages (n = 30 for each group). Data were analyzed by Tukey’s-test. Values represent the means ± SD of three independent experiments (*: p < 0.05; **: p < 0.01). (C) Immunostaining for TK (red) in the middle region of midguts from *C*. *vestalis*-parasitized and nonparasitized 3L *P*. *xylostella* larvae. Nuclei were labeled by DAPI (blue). Scale bars: 50μm. (D) The number of TK-labeled cells in the middle region of midguts from *C*. *vestalis*-parasitized and nonparasitized 3L *P*. *xylostella* larvae (n = 8). Data were analyzed by Student’s t-test. Values represent the means ± SD of more than three independent experiments (*: p < 0.05).

Similar to the results in other insect species [49], *PxTKs* were found to be involved in the lipid metabolism of *P*. *xylostella* larvae, and we wondered whether *C*. *vestalis* parasitization could affect the expression of the PxTK precursor in host larvae. To elucidate this hypothesis, we performed qRT-PCR analysis of the mRNA expression profiles of *PxTK* in the parasitized host larvae and nonparasitized larvae as a control. The results show that the mRNA levels of *PxTK* were significantly upregulated in the parasitized larvae compared to those in the control larvae at all four stages (Fig 4A). We also examined the *PxTK* expression profiles in the midgut of *P*. *xylostella* at the 3L, 4E, 4M and 4L stages. The level of *PxTK* was highly expressed in the parasitized host midgut compared with the control, revealing that parasitization can increase *PxTK* expression in the host midgut (Fig 4B). Moreover, we dissected midguts from 3^rd^ late instar *P*. *xylostella* larvae and stained them with a PxTK antibody. The results showed that the number of EEs that produced PxTK was significantly increased in the parasitized host midguts than in the control midguts (Fig 4C and 4D). Thus, *C*. *vestalis* parasitization leads to an increase in PxTK by upregulating the number of tachykinin-producing EEs in the midgut.

### Reduction of host systemic lipids is important for wasp development

**Fig 5:**
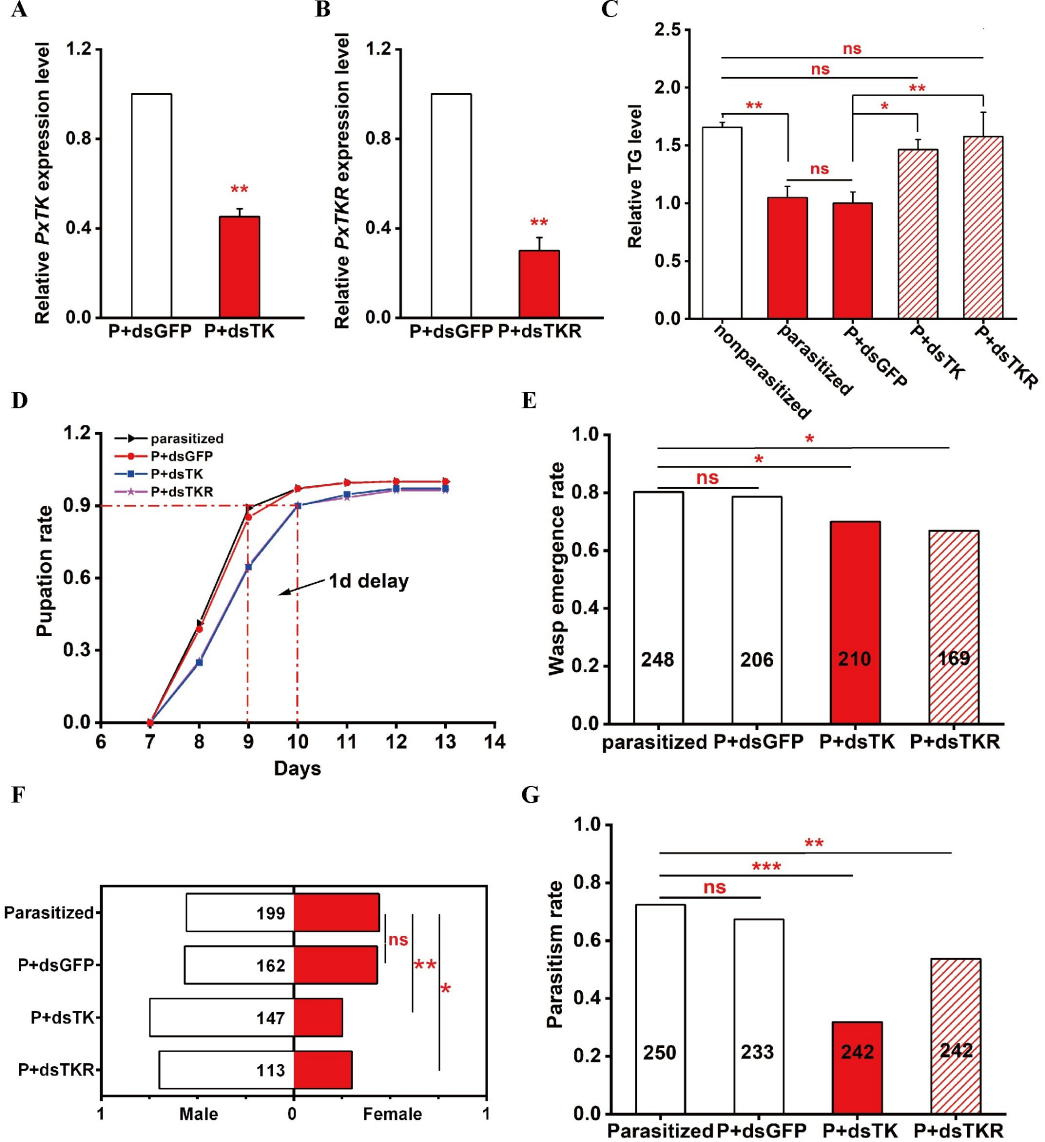
Elevation of host lipids influences the development of wasp offspring and the subsequent parasitic ability. (A) Relative mRNA levels of *PxTK* in parasitized host larvae at 3 days post *PxTK* silencing with dsGFP treatment as a control (n = 5 for each group). Data were analyzed by Student’s t-test. Values represent the means ± SD of three independent experiments (**: p < 0.01). (B) Relative mRNA levels of *PxTKR* in parasitized host larvae at 3 days post *PxTKR* silencing with dsGFP treatment as a control (n = 5 for each group). Data were analyzed by Student’s t-test. Values represent the means ± SD of three independent experiments (**: p < 0.01). (C) Relative levels of triglycerides (TGs) in nonparasitized, *C*. *vestalis*-parasitized, *C*. *vestalis*-parasitized plus dsPxTK-treated (P+dsTK), *C*. *vestalis*-parasitized plus dsPxTKR-treated (P+dsTKR) and *C*. *vestalis*-parasitized plus dsGFP-treated (P+dsGFP) *P*. *xylostella* larvae at 3 days post microinjection (n = 10 for each group). Data were analyzed by Tukey’s-test. Values represent the means ± SD of three independent experiments (*: p < 0.05; **: p < 0.01; ns: not significant). (D) The pupation rate of *C*. *vestalis* in dsPxTK-treated (blue curve, n = 210), dsPxTKR-treated (purple curve, n = 169), dsGFP-treated (red curve, n = 206) and nontreated (black curve, n = 248) *P*. *xylostella* larvae. (E) The wasp emergence rate of *C*. *vestalis* in dsPxTK-treated (n = 210), dsPxTKR-treated (n = 169), dsGFP-treated (n = 206) and nontreated (n = 248) *P*. *xylostella* larvae. Data were analyzed by 2X2 chi-square-test (*: p < 0.05; ns: not significant). (F) The male: female ratios of *C*. *vestalis* that emerged from dsPxTK-treated (n = 147), dsPxTKR-treated (n = 113), dsGFP-treated (n = 162) and nontreated (n = 199) *P*. *xylostella* larvae. Data were analyzed by the 2X2 chi-square test (*: p < 0.05; **: p < 0.01; ns: not significant). (G) The parasitism rates of female *C*. *vestalis* that emerged from dsPxTK-treated (n = 242), dsPxTKR-treated (n = 242), dsGFP-treated (n = 233) and nontreated (n = 250) *P*. *xylostella* larvae. Data were analyzed by the 2X2 chi-square test (*: p < 0.05; **: p < 0.01; ***: p < 0.001; ns: not significant).

To determine the importance of the reduction of host systemic lipids during parasitism, we microinjected dsRNA of *PxTK* and *PxTKR* into parasitized 4E host larvae with *dsGFP* as the control. The *PxTK* and *PxTKR* mRNA levels were significantly decreased after dsRNA injection in the parasitized host (Fig 5A and 5B). We found that the reduction in host whole-body TG levels due to parasitism was largely rescued in the hosts after *PxTK* and *PxTKR* dsRNA treatments (Fig 5C). Most importantly, while 90% of the wasp larvae took approximately 9 days to egress from the control hosts to spin a cocoon and pupate, larvae from the parasitized hosts treated with *dsPxTK* and *dsPxTKR* exhibited a 1-day delay in pupation (Fig 5D). We also found that the wasp emergence rate was significantly lower in the parasitized hosts after treatment with *dsPxTK* and *dsPxTKR* (Fig 5E). Additionally, the ratio of female emerged wasp offspring was reduced when the lipid level was “normal” in the parasitized *dsPxTK-* and *dsPxTKR*-treated hosts (Fig 5F). Due to the immune responses of parasitized hosts, wasp offspring may not all survive. Indeed, approximately 20% of the wasp offspring failed to emerge in control hosts, and most of these died in their early pupal stage. However, more wasp offspring died in *dsPxTK-* and *dsPxTKR-*treated hosts, and the deaths tended to occur in their late pupal stage, when the reduction in systemic lipids was rescued to normal (S10 Fig). Sex is easy to identify in the late pupal stage, and we found that more female wasps were dead than males in the *dsTK-* and *dsTKR*-treated hosts, which might explain why the ratio of emerged female wasp offspring was reduced. It will be both interesting and necessary to investigate the fitness-related effects of host lipid levels for wasps in more depth in future studies.

To determine whether the parasitic ability of female wasp offspring was affected by the host lipid levels, we used 3-day-old well-mated female *C*. *vestalis* that emerged from *dsPxTK-* and *dsPxTKR*-treated hosts and from *dsGFP-*treated controls to parasitize the *P*. *xylostella* larvae. The results show that wasps emerging from *dsPxTK-* or *dsPxTKR*-treated hosts have reduced parasitic efficiency (Fig 5G), indicating that fecundity is largely impaired.

Taken together, these results suggest that a reduction in host lipids during parasitism is necessary for the accurate timing of wasp development, the survival of female wasps, and their subsequent parasitic efficiency.

### CvBV is responsible for *PxTK*-mediated lipid reduction in parasitized *P*. *xylostella* larvae

**Fig 6:**
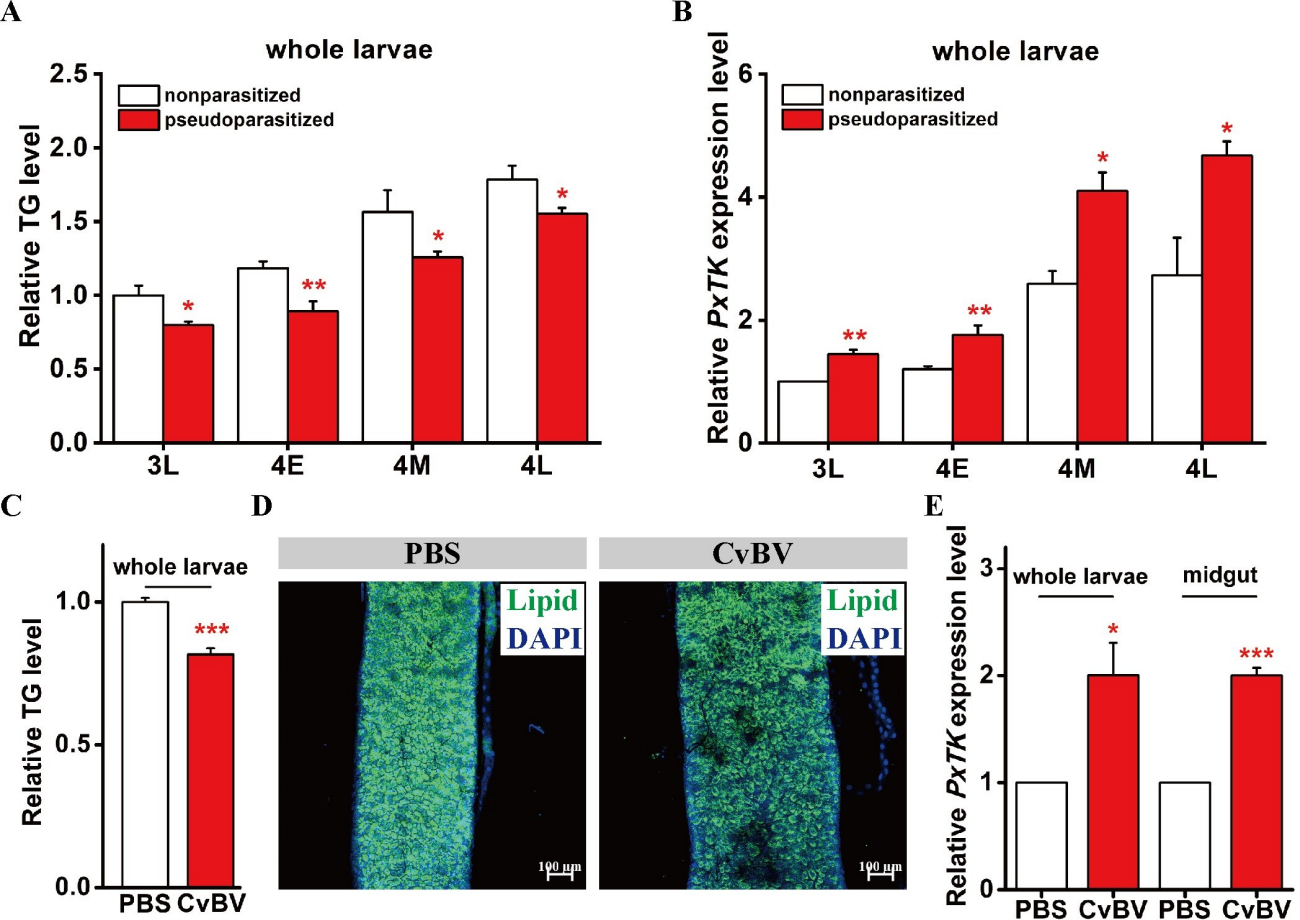
Symbiotic bracovirus affects host lipid levels. (A) Levels of triglycerides (TGs) in *C*. *vestalis*-pseudoparasitized and nonparasitized host larvae at different developmental stages (n = 10 for each group). Data were analyzed by Tukey’s test. Values represent the means ± SD of three independent experiments (*: p < 0.05; **: p < 0.01). 3L: Late 3^rd^ instar; 4E: Early 4^th^ instar; 4M: Middle 4^th^ instar; 4L: Late 4^th^ instar. (B) Relative mRNA levels of *PxTK* in *C*. *vestalis*-pseudoparasitized and nonparasitized host larvae at different developmental stages (n = 5 for each group). Data were analyzed by Tukey’s-test. Values represent the means ± SD of three independent experiments (**: p < 0.01). (C) Levels of TG in 3L *P*. *xylostella* larvae after CvBV injection at a dose of 0.05 FE (female equivalents) per host larva (n = 10 for each group). Data were analyzed by Student’s t-test. Values represent the means ± SD of three independent experiments (**: p < 0.01). (D) Fluorescent images of midguts from *P*. *xylostella* larvae with or without CvBV injection. Lipids were stained with BODIPY (green), and nuclei were labeled with DAPI (blue). Scale bars: 100 μm. (E) Relative mRNA levels of *PxTK* in whole larvae and midguts from *P*. *xylostella* larvae with or without CvBV injection (n = 5 for each group). Data were analyzed by Student’s t-test. Values represent the means ± SD of three independent experiments (*: p < 0.05; ***: p < 0.001).

*C*. *vestalis* has three parasitic factors, namely, venom and a symbiotic *C*. *vestalis* bracovirus (CvBV) that are injected into the host with the egg during oviposition and a special group of cells called teratocytes that are produced during embryogenesis and are released into the host when the *C*. *vestalis* eggs hatch [22,65,66]. To identify which parasitic factors were responsible for the lipid reduction of the parasitized *P*. *xylostella* larvae, we measured the TG contents of pseudoparasitized host larvae. In pseudoparasitization, irradiated female wasps (cobalt-60 for 1 h at a dosage of 100 Gy) lay eggs that are unable to hatch; therefore, analysis of pseudoparasitized hosts allows exclusion of the function of teratocytes. Interestingly, we found that the pseudoparasitized host had a lipid reduction in the 3L, 4E, 4M and 4L stages (Fig 6A), similar to the observations in *P*. *xylostella* larvae parasitized by normal *C*. *vestalis* (Fig 1B). Then, we analyzed the *PxTK* expression levels in pseudoparasitized *P*. *xylostella* at different immature stages. Corresponding to TG reduction, *PxTK* levels were upregulated (Fig 6B), which was also similar to the normally parasitized host (Fig 1C). Overall, these results suggest that venom and/or CvBV, but not teratocytes, may have an effect on host systemic lipid reduction by upregulating *PxTK* expression.

We next injected *C*. *vestalis* venom and CvBV at a dosage of 0.05 FE (female equivalents) per host larva, dosages mimicking those in the real parasitic process [67]. We found a reduction in host lipids and an elevation in *PxTK* levels at 24 hours after CvBV injection (Fig 6C, 6D and 6E), and whereas venom failed to induce any changes (S11 Fig). Collectively, these results suggest that *C*. *vestalis* bracovirus, but not venom, is responsible for *PxTK*-mediated lipid reduction in parasitized *P*. *xylostella* larvae.

### *CvBV 9–2* is necessary for lipid reduction in parasitized *P*. *xylostella* larvae

**Fig 7:**
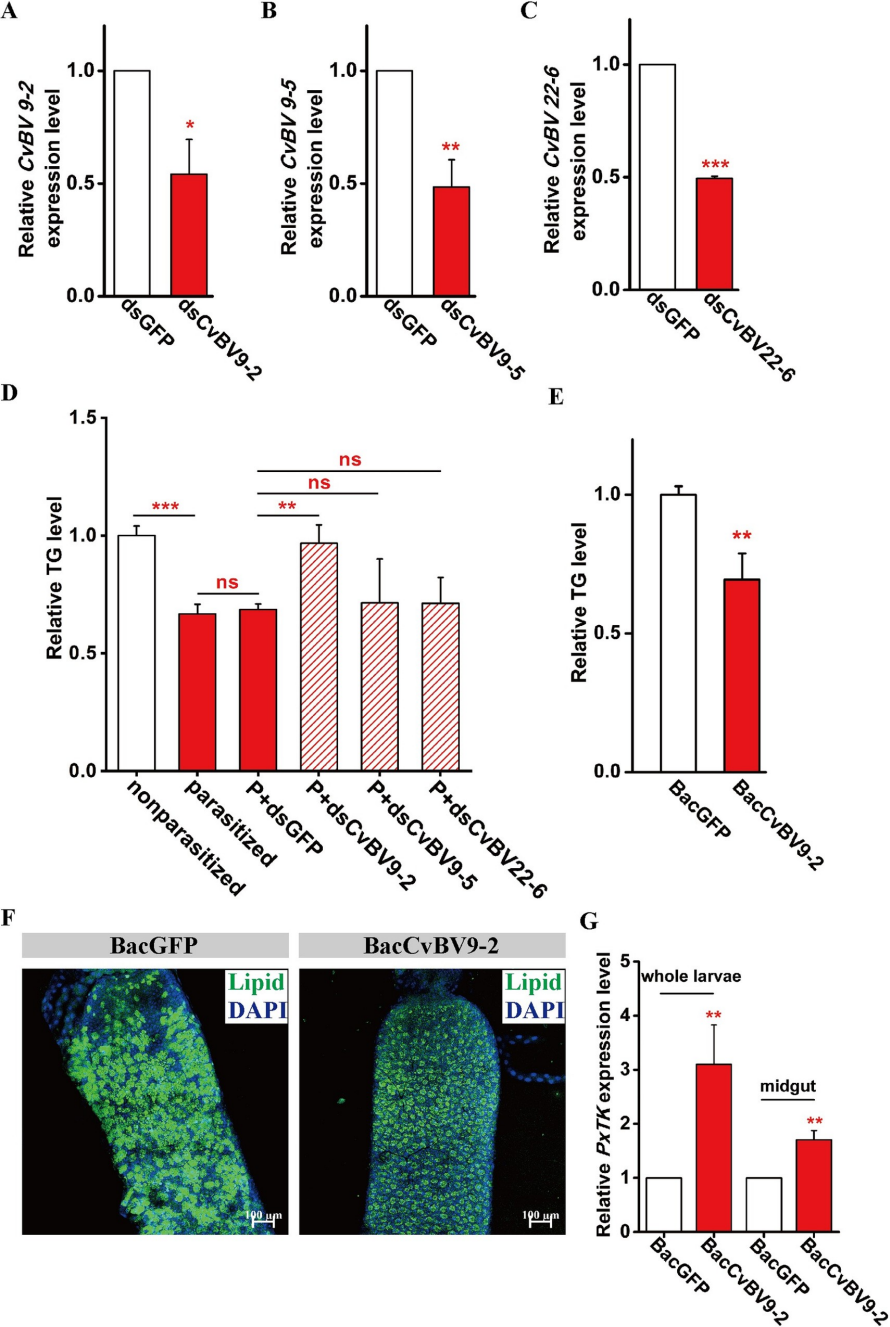
A bracovirus gene, *CvBV 9–2*, is responsible for host lipid level reduction. (A) Relative mRNA levels of *CvBV 9–2* in parasitized *P*. *xylostella* larvae at 1 day post *CvBV 9–2* silencing with dsGFP treatment as a control (n = 5 for each group). Data were analyzed by Student’s t-test. Values represent the means ± SD of three independent experiments (*: p < 0.05). (B) Relative mRNA levels of *CvBV 9–5* in parasitized *P*. *xylostella* larvae at 1 day post *CvBV 9–5* silencing with dsGFP treatment as a control (n = 5 for each group). Data were analyzed by Student’s t-test. Values represent the means ± SD of three independent experiments (**: p < 0.01). (C) Relative mRNA levels of *CvBV 22–6* in parasitized *P*. *xylostella* larvae at 1 day post *CvBV 22–6* silencing with dsGFP treatment as a control (n = 5 for each group). Data were analyzed by Student’s t-test. Values represent the means ± SD of three independent experiments (***: p < 0.001). (D) Relative levels of triglycerides (TGs) in nonparasitized, *C*. *vestalis*-parasitized, *C*. *vestalis*-parasitized plus dsCvBV 9-2-treated (P+dsCvBV9-2), *C*. *vestalis*-parasitized plus dsCvBV 9-5-treated (P+dsCvBV9-5) and *C*. *vestalis*-parasitized plus dsGFP-treated (P+dsGFP) *P*. *xylostella* larvae 1 day post injection (n = 15 for each group). Data were analyzed by Tukey’s-test. Values represent the means ± SD of three independent experiments (*: p < 0.05; **: p < 0.01; ns: not significant). (E) Relative levels of TG in *P*. *xylostella* larvae 1 day post injection with BacCvBV 9–2 and BacGFP (control) (n = 15 for each group). Data were analyzed by Student’s t-test. Values represent the means ± SD of three independent experiments (*: p < 0.05). (F) Fluorescent images of midguts from *P*. *xylostella* larvae 1 day post injection with BacCvBV 9–2 and BacGFP. Lipids were stained with BODIPY (green), and nuclei were labeled with DAPI (blue). Scale bars: 100μm. (G) Relative mRNA levels of *PxTK* in the whole body and midgut of *P*. *xylostella* larvae 1 day post injection with BacCvBV 9–2 and BacGFP, respectively (n = 5 for each group). Data were analyzed by Student’s t-test. Values represent the means ± SD of three independent experiments (**: p < 0.01).

Whole-transcriptome RNA-seq of the midgut of *C*. *vestalis*-parasitized host larvae at 3L, 4E and 4M revealed a total of 63 CvBV genes expressed in all three stages, and differential expression analysis identified three genes, *CvBV 22–6*, *CvBV 9–5* and *CvBV 9–2*, that were highly expressed at all timepoints (S12 Fig). The BLAST P results showed that the three viral genes were highly conserved in the PDVs of diverse *Cotesia* wasp species. Most importantly, none of the three genes have any conserved domains, and their functions are largely unknown. We next used qPCR to detect the expression profiles of these viral genes in different host tissues. The qPCR results confirmed the above transcriptome data showing that *CvBV 22–6*, *CvBV 9–5* and *CvBV 9–2* had high expression levels in host guts (S13 Fig). In addition, *CvBV 22–6* was also highly expressed in the tissues of hemocytes and testes. We further used RNAi technology to identify their functions during the parasitism process. As shown in Fig 7A–7C, the mRNA levels of *CvBV 22–6*, *CvBV 9–5* and *CvBV 9–2* were significantly decreased after RNAi compared to the control (*dsGFP*). Strikingly, we observed a dramatic rescue of TG levels in parasitized whole larvae after knocking down *CvBV 9–2*, but not *CvBV 22–6* or *CvBV 9–5* (Fig 7D). To confirm this result, we used the Bac-to-Bac system to microinject the pFastBac-*CvBV 9–2* virion into the 3M host larvae to mimic *CvBV 9–2* exposure during parasitism and found a similar host systemic lipid reduction as in the parasitized larvae. Compared with that of the pFastBac virion-infected control host, the level of *PxTK* was high in the pFastBac-*CvBV 9–2* virion-infected host larvae (Fig 7E, 7F and 7G). Thus, *CvBV 9–2* may directly upregulate *PxTK* expression in the host midgut, which in turn manipulates lipid production in parasitized host larvae.

## Discussion

Host lipids provide the source of energy and essential fatty acids for parasite survival [10,68–70]. Parasitism-induced changes in host lipid metabolism have been widely reported [70–73]. A range of protozoan parasites are well documented in both experimental and clinical infections to alter lipid biosynthesis in mammals [5,10,74]. For example, *Trypanosoma cruzi* infection is associated with decreased serum lipids along with increased low-density lipoprotein (LDL) and cholesterol in tissues [75]. In this study, we have shown that host *P*. *xylostella* lipid homeostasis is largely impaired and host systemic lipid amounts are reduced after *C*. *vestalis* parasitization, indicating that changes in lipid metabolism in response to “infection” are a broad event in both vertebrates and invertebrates.

Lipids are among the most important nutrients to parasitoids and facilitate infection, development and reproduction [59,70]. Parasitoids are generally incapable of de novo lipid synthesis. Instead, they acquire lipid nutrition, usually TGs, directly from their hosts and usually share similar sets of lipid metabolites with their hosts [70,76]. Accumulating evidence has shown that parasitoids can modify host lipid metabolism to promote their development and maturity [77–81]. For example, *Lysiphlebia japonica* parasitization causes a sharp increase in TG levels in the body of the *Aphis gossypii* host, which is necessary for *L*. *japonica* growth and reproduction [80]; similarly, the parasitoid *Chelonus inanitus* also causes an accumulation of lipids in the whole body of the *Spodoptera littoralis* host, which is crucial for parasitoid survival [82,83]. Recently, a lipidomics study revealed that the endoparasitoid wasp *Pteromalus puparum* increases TG levels in different tissues of the parasitized host, such as the fat body and hemolymph [77]. In contrast to these previous reports, we found that parasitized *P*. *xylostella* host larvae exhibit reduced whole-body TG levels at all stages post *C*. *vestalis* infection. Interestingly, the circulating TG in host hemolymph was significantly increased at the 4L stage in parasitized compared to nonparasitized larvae. The differences in manipulation strategies might reflect the different parasitic and feeding strategies of the parasitoids. *C*. *vestalis* is a hemolymph feeder and does not consume all the host resources prior to pupation, which is significantly different from some other parasitoids that have a tissue feeder strategy and consume all the host resources to pupate. Our results also suggest that *C*. *vestalis* needs more TG nutrition in host hemolymph at the 4L stage. Indeed, the *C*. *vestalis* larvae reach their mature stage and egress from the 4L host to spin a cocoon and pupate. Moreover, some studies have shown that wasp parasitization causes hyperlipidemia by lysis of host fat body cells [20,73,84].

TGs are primarily stored within the fat body of hosts and are probably the largest lipid resources for the developing larvae of parasitoids [7,13,70,85]. Although we focused on the principal lipid nutrient, TGs, it is likely that other lipids are also regulated by *C*. *vestalis* infection, because lipids such as sterols and phospholipids are important for insect hormone synthesis and cell membrane structure maintenance [55,86,87]. It will be necessary to utilize novel techniques, such as lipidomics analysis, to comprehensively investigate the changes in all types of lipids in *P*. *xylostella* post wasp infection and to determine the most important lipids in the context of parasitoid-host interactions in future studies.

To determine the mechanisms underlying the reduction in host lipid levels caused by *C*. *vestalis*, we cloned the *PxTK* gene and deduced six mature PxTKs, which were processed and secreted from the midgut enteroendocrine cells of the host *P*. *xylostella* larvae. Using the RNA interference technique, we successfully reduced the expression of *PxTK* and its receptor, *PxTKR*, in host larvae and found that loss of TKs resulted in an increase in lipid production, which was similar to previous reports in *Drosophila*. Then, we microinjected chemically synthesized PxTKs into *P*. *xylostella* larvae and found that exogenous PxTK1 and PxTK3 could induce lipid reduction, but addition of the other four mature PxTKs did not. Most importantly, we found that *C*. *vestalis* parasitization could increase the expression of *PxTK* by upregulating the number of EEs in the midgut. These results suggest that the induction of *PxTK* in the *P*. *xylostella* midgut is required for systemic lipid reduction upon *C*. *vestalis* parasitization. Our study also reveals that development and subsequent parasitic efficiency were impaired for wasps emerging from *dsPxTK-* and *dsPxTKR* RNAi-treated hosts whose systemic lipid levels were higher than normal. It is likely that PxTK and PxTKR are involved in other physiological processes in *P*. *xylostella* larvae, as we have shown that TK was also highly expressed in the CNS and some other tissues. As such, specifically knocking down the PxTK and PxTKR genes in EEs in the host midgut instead of the systemic knockdown performed in this study would be a more accurate approach to investigate whether the observed effects on the parasitoid are due to altered TG levels alone. However, unlike *D*. *melanogaster*, *P*. *xylostella* is not a model insect, and reagents for knocking down genes in a specific tissue are lacking.

*C*. *vestalis* has several weapons that allow it to manipulate host energy metabolism, including venom, CvBV and teratocytes [22,65,66]. CvBV virions assemble in the ovary calyx of female wasps and are injected into *P*. *xylostella* larvae along with the wasp eggs during oviposition. The virions have been found to infect most host immune cells and some other tissue cells [31,67]. Here, we show that CvBV could successfully infect *P*. *xylostella* midgut cells. Our transcriptome data revealed that 63 CvBV genes were expressed in the infected host midgut, with three genes, *CvBV 22–6*, *CvBV 9–5* and *CvBV 9–2*, having consistently high expression profiles. Our functional analysis further indicated that only *CvBV 9–2* is necessary to lower systemic lipid levels in parasitized *P*. *xylostella* larvae. Moreover, *CvBV 9–2* is responsible for the elevation of *PxTK* expression in the midgut EEs of the parasitized host. However, we only tested the function of these three genes with higher expression levels in this study, and we cannot rule out the possibility that some other CvBV genes might also affect host systemic lipid levels. Whether *CvBV 9–2* is the key causative agent remains to be tested.

**Fig 8:**
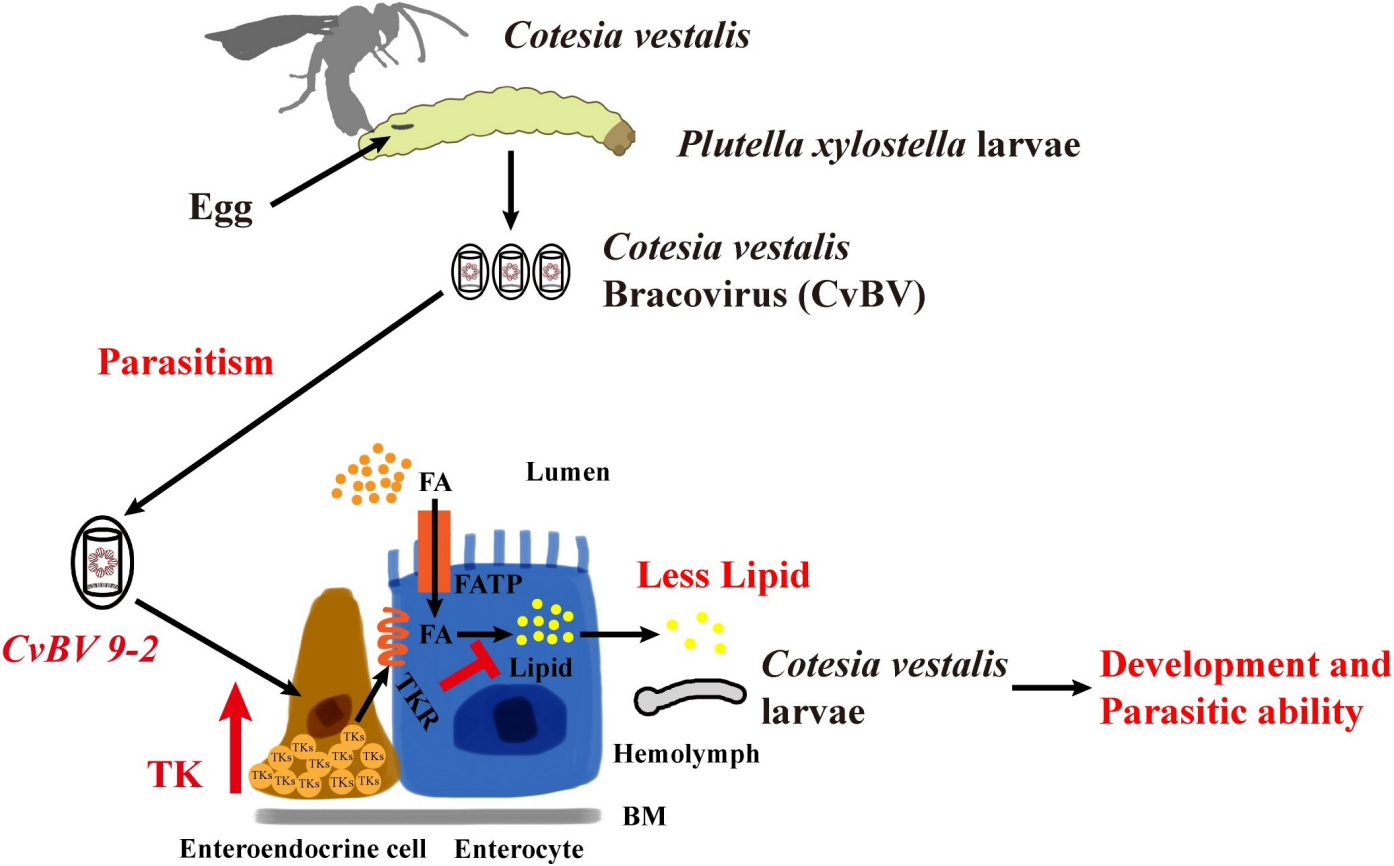
Model for manipulation of host tachykinin signaling and lipid metabolism by a *C*. *vestalis* bracovirus gene, *CvBV 9–2*. Schematic diagram of a model for the processes by which a *C*. *vestalis* bracovirus gene, *CvBV 9–2*, reduces the lipid level of *P*. *xylostella* larvae by upregulating intestinal TK expression. During parasitism, *C*. *vestalis* bracovirus is injected into *P*. *xylostella* larvae along with the eggs and then infects the different host tissues. A bracovirus gene, *CvBV 9–2*, is responsible for the induction of *PxTK* in enteroendocrine cells of the host midgut. Thus, the increase in *PxTK* restrains lipid production in enterocyte cells through the PxTK receptor. The reduction in host lipids during parasitism is important for the development of *C*. *vestalis* larvae, female wasp survival and subsequent parasitic efficiency.

In conclusion, we discovered a strategy in which parasites interact with host midgut EEs to reduce host lipid production, which is necessary for parasite development and parasitic ability, via upregulation of PxTK signaling. The polydnavirus gene *CvBV 9–2* might cause the overexpression of PxTKs in the host midgut (Fig 8). These findings provide innovative insights into the mechanisms by which parasitic wasps manipulate host lipid homeostasis and may help to expand our knowledge of other parasitic systems and develop therapeutic strategies for human diseases, including malaria, trypanosomiasis, leishmaniosis and other human parasitic protozoa-caused diseases.

## Materials and methods

### Insects

The *P*. *xylostella* and *C*. *vestalis* used in this study were reared as previously described [22]. Briefly, *P*. *xylostella* was reared on cabbage at 25 ± 1°C, 65% ± 5% relative humidity, and a 14 hour light:10 hour dark photoperiod. *C*. *vestalis* was reared by allowing adult females to parasitize mid-3^rd^ instar (3M) *P*. *xylostella* larvae. For pseudoparasitism, female *C*. *vestalis* adults were irradiated with cobalt-60 for 1 hour at a dose of 100 Gy. All adult wasps were fed on a 20% honey/water (V/V) solution.

### Cell lines

Human embryonic kidney 293 (HEK293) cells were maintained at 37°C in 5% CO_2_ in basic DMEM (Gibco) plus 10% fetal bovine serum (FBS, Gibco). *Spodoptera frugiperda* (Sf-9) insect cells were maintained in 100 mm culture dishes in Sf900 II serum-free medium (Invitrogen) at 27°C under ambient atmosphere.

### Subcloning of *PxTK*, *PxTKR* and CvBV genes

Nonparasitized or parasitized *P*. *xylostella* larvae were homogenized, and total RNA was extracted with the Total RNA Extraction Reagent Kit (Vazyme) according to the manufacturer’s instructions. The quality and concentration of the isolated total RNA were estimated by electrophoresis and a NanoDrop 2000 (Thermo Fisher Scientific). Then, complementary DNA was synthesized using a PrimeScript 1^st^ Strand cDNA Synthesis Kit (Takara) according to the manufacturer’s instructions. The entire coding regions of the *PxTK*, *PxTKR* and CvBV genes were cloned and inserted into the pGEM-T Easy Vector (Promega). All constructs were sequenced to verify the identity of the sequences. The primers used are listed in S1 Table.

### Peptide synthesis, injection and antibody preparation

*P*. *xylostella* tachykinin peptides (PxTKs) were synthesized by Sangon Biotech, and their sequences are listed in S2 Table. Purification of PxTKs was performed by reversed-phase high-performance liquid chromatography (HPLC) using a 30-min linear gradient of 100% acetonitrile and 0.1% trifluoroacetic acid at a flow rate of 1.0 ml/min. Elution was monitored by absorbance at 214 nm, and the purities were ≥95%. The molecular weight of each peptide was measured by mass spectrometry.

To confirm the function of each PxTK peptide, approximately 1 ng of each peptide and their mixture were injected into mid-3^rd^ instar (3M) *P*. *xylostella* larvae per day. The peptide injections were performed once per day for 3 consecutive days. After injections, the *P*. *xylostella* larvae were homogenized, and the samples were prepared for detection of the lipid contents.

The polyclonal antibodies used in this study were designed and synthesized by ABclonal Technology Company. Briefly, the anti-PxTKR antibody was raised against one portion of the C-terminus of PxTKR (CGALDRGGLSRHRAFGPER). The anti-PxTK antibody was raised against PxTK1 (APSGFLGMR). Both peptides were synthesized with N-terminal cysteine (underlined) to enable coupling of maleimide-coupled KLH (keyhole limpet hemocyanin). Then, two rabbits were immunized with each peptide, and the serum was purified by the affinity purification method. The purified antibodies were confirmed by dot blotting at a concentration of 1:1000 (100 ng antigen) before use.

### Preparation of recombinant transfection vectors

Amplified products of *PxTKR* were excised from the pGEM-T Easy Vector and introduced into the pcDNA 3.1^(+)^ vector (Invitrogen) using the restriction sites for expression in mammalian HEK293 cells. The primers used for PCR are shown in S1 Table. The Kozak sequence (GCCACC) was added to the forward primer of *PxTKR*, allowing efficient, high-level expression of the recombinant protein in HEK293 cells. The recombinant vectors were confirmed by PCR using a pair of universal primers (S1 Table).

To generate the expression vectors, *CvBV 9-2-* and *GFP*-amplified products were cloned from the pGEM-T Easy Vector and introduced into the pFastBac HTB vector (Invitrogen) using homologous recombination sites (ClonExpress II One Step Cloning Kit, Vazyme) according to the manufacturer’s instructions. The primer sequences used for PCR are shown in S1 Table. *E*. *coli* DH10 cells (Invitrogen) were transformed with the above plasmids and helper plasmids to generate recombinant bacmids encoding the CvBV 9–2 and GFP viruses. The recombinant bacmids were confirmed by PCR using a pair of universal primers (S1 Table). All constructs were sequenced to verify the correct sequences and orientations.

### Cell transfection

To establish a stable HEK293/PxTKR cell line, HEK293 cells were seeded in 30 mm culture dishes (to 80% confluence) and transfected with 2.5 μg of PxTKR/pcDNA 3.1^(+)^ plasmid using a Lipofectamine 3000 Kit (Invitrogen) according to the manufacturer’s instructions. Forty-eight hours later, the transfected cells were reseeded in 100 mm dishes and selected with DMEM plus 10% fetal bovine serum and G418 (Invitrogen) at a final concentration of 800 mg/L. Three weeks later, the cells that were supposed to stably express *PxTKR* were confirmed by PCR using the HindIII-PxTKR-F and Xhol-PxTKR-R primers in S1 Table and sequenced.

To obtain the CvBV 9–2 and GFP viruses, the Bac-to-Bac Baculovirus Expression Vector System (Invitrogen) was used according to the manufacturer’s instructions. Briefly, Sf-9 cells were seeded in a 6-well format with 2 ml Sf900 II serum-free medium. The Sf-9 cells were considered ready to use when they were in the log phase (1.5–2.5 × 10^6^ cells/ml) with greater than 95% confluence. Then, 8 μl Cellfectin II, 1 μl baculovirus DNA and 200 μl Sf900 II serum-free medium were added together and gently vortexed to ensure complete mixing. The DNA-lipid mixture was incubated for 15–30 minutes at room temperature, added to Sf-9 cells and incubated at 27°C for 3–5 hours. Finally, the transfection mixture was removed and replaced with 2 ml Sf900 II serum-free medium, and the cells were incubated at 27°C for another 72 hours.

### Bacmid purification and injection

After transfection, the medium containing virus was transferred to sterile 15-ml snap-cap tubes and centrifuged at 800 × g for 10 minutes at 4°C to remove cells and large debris. The pure supernatant containing virus was transferred to 1.5 ml tubes and centrifuged at 20,000 × g for 1 hour to obtain a high concentration of CvBV 9–2 or GFP virus. In each experiment, approximately 10^4^ copies of virus were injected into 3M *P*. *xylostella* larvae. The titer of each virus was determined by qPCR. RNA samples of homogenates from whole bodies or midguts were extracted at 1 day post injection to detect the expression level of *PxTK*. In addition, at 1 day after injection, the midguts or whole bodies of host *P*. *xylostella* larvae were homogenized to assay the lipid contents.

### cAMP assays

The cAMP assays were performed as previously described using the cAMP-Glo assay kit (Promega). Briefly, HEK293/PxTKR cells were aliquoted into a 96-well plate at a density of 10,000 and then incubated overnight at 37°C in 5% CO_2_. Subsequently, the cells were treated with cAMP stabilizers as follows: 0.5 mM 3-isobutyl-1-methylxanthine buffer (IBMX) (Sigma-Aldrich, I5879; dissolved in 1x PBS, pH 7.2) alone for 15 minutes; 10 μM forskolin (Sigma-Aldrich, F6886; dissolved in 1x PBS, pH 7.2) plus 0.5 mM IBMX for 15 minutes; or 10 pM to 10 μM (10^−11^–10^−4^ mol/L, serials of ten times dilution in 1x PBS, pH 7.2) of each individual PxTK plus 0.5 mM IBMX for 15 minutes, all at room temperature. After stimulation, cells were processed for the cAMP Glo assay as per the manufacturer’s instructions (Promega, V1502). Luminescence was measured using a microplate reader (BIO-RAD). The luminescence signal of stimulated cells was subtracted from that of cells exposed to 1x PBS (with 0.5 mM IBMX), and the ranges of the cAMP response of HEK293/PxTKR cells to all concentrations of PxTKs to forskolin were calculated. Nonlinear fitting curves were produced using GraphPad Prism 8.3.

### Expression analysis by qPCR

To analyze *PxTK* expression levels in different instar *P*. *xylostella* larvae, RNA samples of overall homogenates were extracted individually from mid-3^rd^ instar (3M) larvae, late-3^rd^ instar (3L) larvae, early-4^th^ instar (4E) larvae, mid-4^th^ instar (4M) larvae and late-4^th^ instar (4L) larvae. To analyze *PxTK* expression levels in different tissues of the 4L larvae, hemolymph was collected by bleeding the 4L larvae from a cut preleg, and the epidermis, silk gland, fat body, midgut, central nervous system (CNS), Malpighian tubule (MT), and testis were dissected and collected under a dissecting microscope (ZEISS) in 1X PBS. RNA was extracted from different tissues. cDNA was prepared from the extracted RNAs using ReverTra Ace qPCR RT Master Mix with a gDNA Remover kit (Toyobo) according to the manufacturer’s instructions. qPCR reactions were conducted on a CFX Connect real time system (BIO-RAD) using THUNDERBIRD qPCR Mix (Toyobo). Each qPCR was performed for at least three biological replicates under the following conditions: 95°C for 60 sec, followed by 40 cycles of 95°C for 15 sec and 60°C for 30 sec. The *Px-β-Tubulin* gene (GenBank accession No. EU127912) and *Px-β-Actin* gene (GenBank accession No. NM_001309101) of *P*. *xylostella* were used as internal controls. The primer sequences used for qPCR analysis are shown in S1 Table. The relative expression levels were analyzed using the 2^-ΔΔCt^ method.

### CvBV and venom collection, and injection

To assess whether CvBV and/or venom are responsible for the decreased lipid levels in parasitized *P*. *xylostella* larvae, CvBV and venom were collected from female wasps as previously described. The ovaries of 4-day-old *C*. *vestalis* female wasps were dissected in 1X PBS on ice, and the calyxes were punctured individually. The calyx fluid was filtered using a 0.22 μm filter to remove cellular debris and centrifuged at 20,000 × g for 1 hour. The viral particle pellet was resuspended in PBS. The venom reservoirs of 4-day-old *C*. *vestalis* female wasps were dissected in 1X PBS on ice, and the reservoirs were punctured individually. The venom fluid was centrifuged at 2,000 × g for 10 min to remove cellular debris. All samples were stored in a −80°C refrigerator for further usage. The viral particles and venom fluid collected from a single female adult was defined as one FE (female equivalent). To infect host larvae, 0.05 FE CvBV particles or venom was injected into 3M *P*. *xylostella* larvae. RNA samples of overall homogenates or midguts were extracted individually at 1 day post injection to detect the expression level of *PxTK*. In addition, at 1 day after injection, the midgut or whole body of *P*. *xylostella* larvae were homogenized to assay the lipid contents.

### RNA interference

For RNAi, 200~500 bp DNA fragments of the target genes were PCR amplified from the relevant plasmids as templates. Forward and reverse primers containing T7 promoter sequences at their 5’ end were used for PCR amplification of the double-stranded RNA (dsRNA) templates (S1 Table). dsRNA was synthesized using a T7 RiboMAX Express Kit (Promega) according to the manufacturer’s instructions. To avoid off-target effects, two nonoverlapping dsRNAs were synthesized for *PxTK*. The length of the *PxTK* open reading frame (ORF) is 891 bp, the first dsRNA that we designed for *PxTK* silencing is from the 85 bp position to the 600 bp position on the *PxTK* ORF (dsPxTK), and the second dsRNA for *PxTK* silencing is from the 604 position to the 877 bp position (dsPxTK_604-877_). However, one dsRNA was synthesized for *PxTKR* silencing in this study (dsPxTKR). A 328-bp coding sequence from green fluorescent protein (GFP) was used as a control dsRNA (dsGFP). The dsRNA was quantified using a NanoDrop 2000 spectrophotometer (Thermo Scientific). A total of 1 μg dsRNA was injected into each *P*. *xylostella* larva using the Eppendorf FemtoJet 4i Microinjector with the following parameters: injection pressure = 900 hPa; injection time = 0.15 sec. At least three biological replicates were performed.

### TG content measurements

TG measurements were performed as previously described. Briefly, whole bodies, hemolymph and midguts were collected and homogenized in 1X PBS with 0.1% Triton. All the samples were centrifuged at 14,000 rpm for 10 minutes, and the supernatants were heated at 70°C for 5 minutes. The TG content was measured by using Serum TG determination kits (Sigma) and was normalized to the protein amounts in each homogenate, which were measured by using Bradford Reagent (Invitrogen). Experiments were repeated at least three times.

### Lipid staining, immunostaining and microscopy

For lipid staining, *P*. *xylostella* larvae were dissected in 1X PBS, and the midguts were fixed in 4% formaldehyde in 1X PBS for 20 minutes. After fixation, the samples were washed with 1X PBST (1X PBS containing 0.1% Triton) 3 times for 5 minutes each. The samples were incubated for 30 minutes in a 1:1000 dilution of 1 mg/ml BODIPY 493/503 (Invitrogen) in 1X PBS and then rinsed twice with 1X PBS. Stained samples were mounted in ProLong Gold Antifade Mountant with DAPI (Invitrogen).

For immunostaining, *P*. *xylostella* larvae were dissected in 1X PBS, and the midguts were fixed in 4% formaldehyde in 1X PBS for 20 minutes. Fixed tissues were washed 3 times with 1X PBST for 10 minutes each and then blocked in 1% BSA for 3 hours at room temperature. Samples were incubated with primary antibody overnight at 4°C and washed 3 times with 0.1% PBST for 5 minutes per wash. Samples were then incubated in secondary antibody for 1 hour at room temperature and washed 3 times with 1X PBST for 5 minutes each. Tissues were mounted in ProLong Gold Antifade Mountant with DAPI (Invitrogen). All experiments were repeated at least three times. Fluorescent images were captured by Zeiss LSM 800 laser confocal microscopy and edited using ImageJ (NIH) software.

### Transcriptome analysis

The midguts of the 3L, 4E and 4M instar *P*. *xylostella* larvae were dissected under a microscope in 1X PBS on ice. Total RNA was isolated using TRIzol reagent according to the manufacturer’s instructions. The quality and concentration of the isolated total RNA were estimated by electrophoresis and a NanoDrop 2000. The midgut transcriptomes were produced by ANOROAD. cDNA libraries for transcriptome sequencing made from total RNA of 3L, 4E and 4M midguts were prepared using the NEBNext Ultra RNA Library Prep Kit (NEB #E753) for Illumina in conjunction with the NEBNext Poly(A) mRNA Magnetic Isolation Module (NEB #E7490). Libraries were validated and quantified before being pooled and sequenced on an Illumina HiSeq 2000 (Illumina) sequencer with a 200 bp paired-end protocol. Sequences were de novo assembled using Trinity on a Galaxy Portal, and both ends were sequenced. The raw reads were cleaned by removing adaptor sequences, empty reads and low-quality sequences (reads with unknown sequences ‘N’).

### Wasp offspring development and subsequent parasitic rate

Female *C*. *vestalis* wasps were allowed to parasitize *P*. *xylostella* larvae. At 60 hours after infection, the parasitized larvae were microinjected with dsRNAs, including *dsTK*, *dsTKR* and *dsGFP* (control). Then, we recorded the time for wasp pupation and the number of emerged male and female adults to calculate the pupation rate, wasp emergence rate and female/male ratio for each treatment. To assess the parasitic ability of the new wasp, eclosed female *C*. *vestalis* from different groups were used to parasitize 3M *P*. *xylostella* host larvae for 1 hour. After infection, the hosts were kept in a 25°C incubator until the wasps emerged. The parasitic rate was calculated using the following formulas: parasitic rate = (number of hosts–number of emerged *P*. *xylostella*)/number of hosts.

### Statistical analysis

All statistical analyses were performed using SPSS 16.0 software (one-way ANOVA). Data are expressed as the means ± SD, and each treatment had more than three biological replicates. The data, including the gene expression levels and TG contents, were analyzed by Tukey’s test, with a significance threshold of p < 0.05. The data showing the wasp emergence rate, male: female ratio and parasitism rate were analyzed by 2X2 chi square, with a significance threshold of p < 0.05 (* represents p<0.05; ** represents p<0.01; *** represents p<0.001; ns: not significant).

## Supporting information

S1 Fig *C. vestalis* parasitization affects host lipid contents in midguts. Light microscope images of midguts from 3L, 4E, 4M and 4L *C*. *vestalis*-parasitized and nonparasitized *P*. *xylostella* larvae. The different regions of 4L midguts were labeled. Lipid was stained by Oil Red O. Scale bars: 500 μm. 3L: Late 3^rd^ instar; 4E: Early 4^th^ instar; 4M: Middle 4^th^ instar; 4L: Late 4^th^ instar.
(TIF)

S2 Fig Gene structure and deduced amino acid sequence of *P. xylostella* tachykinin. (A) The *P*. *xylostella tachykinin* (*PxTK*) transcript has three exons, and their nucleotide lengths are 73 bp, 855 bp and 116 bp. (B) The underlined amino acids indicate the putative signal peptide of *PxTK*. The gray labeled amino acids indicate the predicted mature PxTK peptides. Predicted amination amino acids (G) with dibasic cleavage sites (combinations of K and R) are in bold italics.
(TIF)

S3 Fig Alignments of TK mature peptides of *P. xylostella* with other insects. A conserved C-terminal motif is identified from each mature peptide with the sequence FxGxR (x is a variable residue). PxTK: *Plutella xylostella* tachykinin peptide; BmTRP: *Bombyx mori* tachykinin-related peptide; DTK: *Drosophila melanogaster* tachykinin peptide.
(TIF)

S4 Fig *P. xylostella* TK is highly expressed in the middle region of the midgut. (A) Immunostaining for TK (red) in the whole midgut from 3L *P*. *xylostella* larvae. Nuclei were labeled by DAPI (blue). Scale bar: 500 μm. The number of TK-labeled cells was greater in the middle region of the *P*. *xylostella* midgut (C) than in the anterior (B) and posterior parts (D). The TK-secreting cells are indicated by arrowheads in B, C and D. Scale bars: 50 μm. 3L: Late 3^rd^ instar.
(TIF)

S5 Fig Cloning and expression of the PxTK receptor in HEK293 cells. *P*. *xylostella* tachykinin receptor (*PxTKR*) was subcloned into the pcDNA 3.1^(+)^ plasmid and then transfected into HEK293 cells. PxTKR was localized on the membrane in HEK293/PxTKR cells but not in HEK293 control cells (anti-TKR, red). Nuclei were labeled by DAPI (blue). Scale bars: 20 μm.
(TIF)

S6 Fig Modulation of intracellular cAMP levels in HEK293 cells stably expressing PxTKR. Relative cAMP levels of HEK293 cells with PxTKR expression when treated with the indicated compounds at a dose of 10 μM. The tested PxTK peptides were TK1, TK2, TK3, TK4, TK5 and TK6. Forskolin was used as a positive control. Data were analyzed by Tukey’s test. Values represent the means ± SD of more than three independent experiments (**: p < 0.01; ***: p < 0.001).
(TIF)

S7 Fig Silencing PxTK results in an elevation of lipid level in *P. xylostella* larvae. (A) Relative mRNA levels of *TK* in *P*. *xylostella* midgut at 3 days post *PxTK*_*604-877*_ dsRNA silencing with dsGFP treatment as a control (n = 5 for each group). Three biological replicates were performed. Data are the means ± SD; significance was determined by Student’s t-test (***: p < 0.001). (B) Relative levels of triglycerides (TGs) from dsPxTK_604-877_- and dsGFP (control)-treated *P*. *xylostella* larvae at 3 days post microinjection (n = 30 for each group). Data were analyzed by Tukey’s test. Values represent the means ± SD of five independent experiments (***: p < 0.001).
(TIF)

S8 Fig The alignment of six mature *P. xylostella* TKs. (TIF)

S9 Fig Microinjection of PxTK1 or PxTK3 into *P. xylostella* larvae could reduce lipid levels. (A) Relative levels of triglycerides (TGs) from the whole body of *P*. *xylostella* larvae at 3 days post microinjection of chemically synthesized PxTK1 at different dosages (n = 5). Data were analyzed by Tukey’s test. Values represent the means ± SD of five independent experiments (*: p < 0.05; **: p < 0.01). (B) Relative levels of TG from the whole body of *P*. *xylostella* larvae at 3 days post microinjection of chemically synthesized PxTK3 at different dosages (n = 5). Data were analyzed by Tukey’s test. Values represent the means ± SD of five independent experiments (**: p < 0.01; ***: p < 0.001).
(TIF)

S10 Fig Mortality is higher in female wasps when tachykinin signaling is knocked down in parasitized *P. xylostella* larvae. The rates of dead pupae in the early pupal stage (white column) and in the late pupal stage (female: column with red dashed lines and male: red column).
(TIF)

S11 Fig Venom is not responsible for *PxTK*-mediated lipid alteration in parasitized *P. xylostella* larvae. (A) Levels of triglycerides (TGs) in *P*. *xylostella* larvae at 1 day post microinjection of venom with a dose of 0.05 FE (female equivalents) (n = 10 for each group). Values represent the means ± SD of five independent experiments. (B) Relative *PxTK* mRNA levels in *P*. *xylostella* larvae at 1 day post microinjection of venom with a dose of 0.05 FE (female equivalents) (n = 10 for each group). Data were analyzed by Student’s t-test. Values represent the means ± SD of three independent experiments. ns: not significant.
(TIF)

S12 Fig Heat map of *C. vestalis* bracovirus genes in the midguts of parasitized *P. xylostella* larvae. The expression profiles of *C*. *vestalis* bracovirus (CvBV) genes in the midguts of parasitized 3L, 4E and 4M host larvae. Red and green colors in the heat map indicate high and low expression levels, respectively. The most highly expressed genes in all tested development stages were *CvBV 22–06*, *CvBV 09–05*, and *CvBV 09–02*. The number before the short dash represents the bracovirus circle, and the number after the short dash represents the order of the genes on that circle. 3L: Late 3^rd^ instar; 4E: Early 4^th^ instar; 4M: Middle 4^th^ instar.
(TIF)

S13 Fig Expression patterns of *CvBV 9–2*, *CvBV 9–5* and *CvBV 22–6* in different tissues of parasitized *P. xylostella* larvae. Relative mRNA levels of *CvBV 9–2*, *CvBV 9–5* and *CvBV 22–6* in six different tissues of *P*. *xylostella* 4L larvae (n = 30). Values represent the means ± SD of three independent experiments. 4L: Late 4^th^ instar.
(TIF)

S1 Table List of primer sequences used in this study. (XLSX)

S2 Table Amino acids sequences of PxTKs used in this study. (XLSX)
