# Supplementary figures and images for: Symbiotic bracovirus of a parasite manipulates host lipid metabolism via tachykinin signaling
Source: PLoS Pathog. 2021 Mar 1;17(3):e1009365. doi: 10.1371/journal.ppat.1009365 (PMC7951984; doi:10.1371/journal.ppat.1009365)

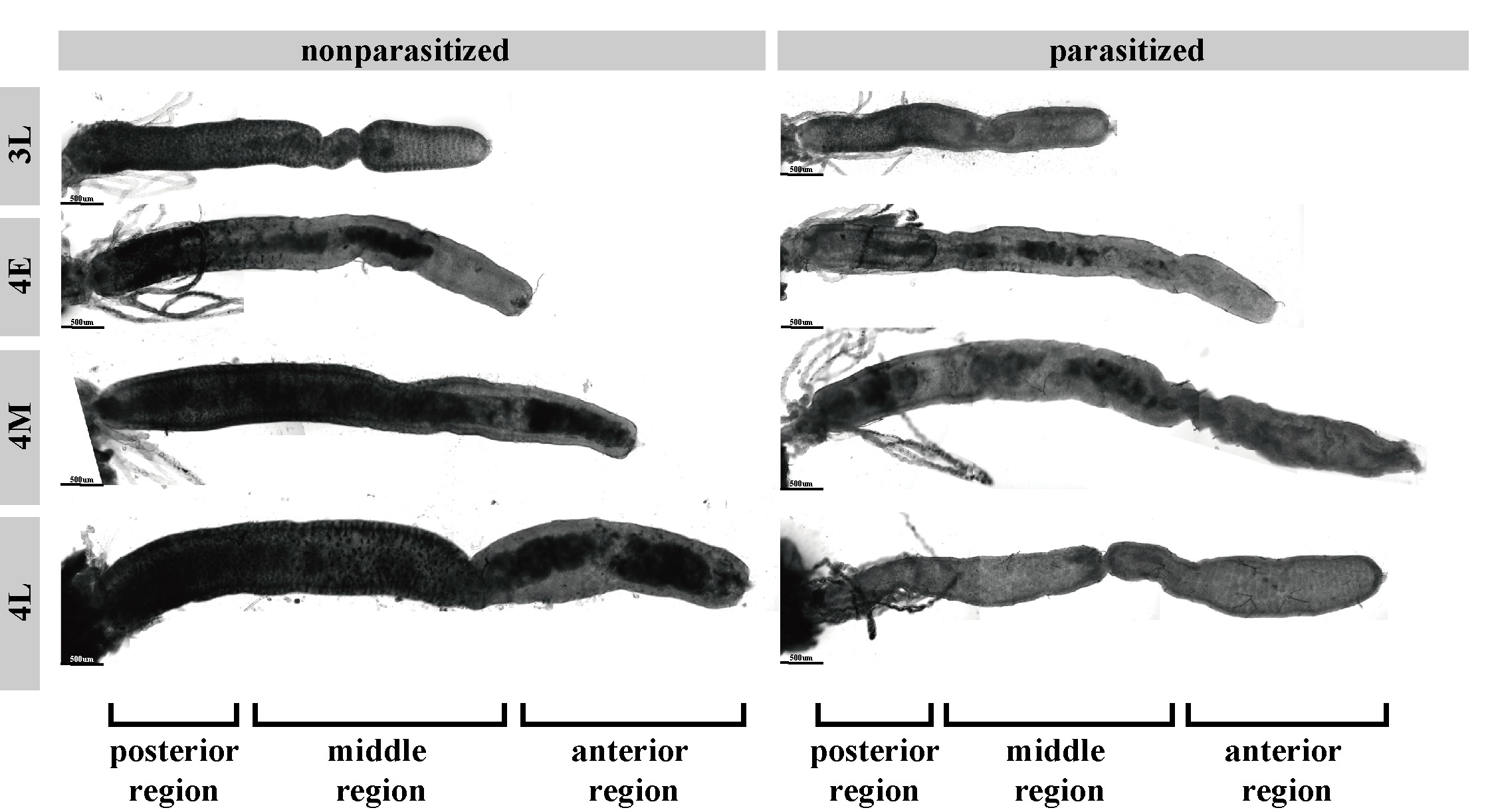

Supplement: S1 Fig — Light microscope images of midguts from 3L, 4E, 4M and 4L C. vestalis-parasitized and nonparasitized P. xylostella larvae. The different regions of 4L midguts were labeled. Lipid was stained by Oil Red O. Scale bars: 500 μm. 3L: Late 3rd instar; 4E: Early 4th instar; 4M: Middle 4th instar; 4L: Late 4th instar. (TIF) [file ppat.1009365.s001.tif]

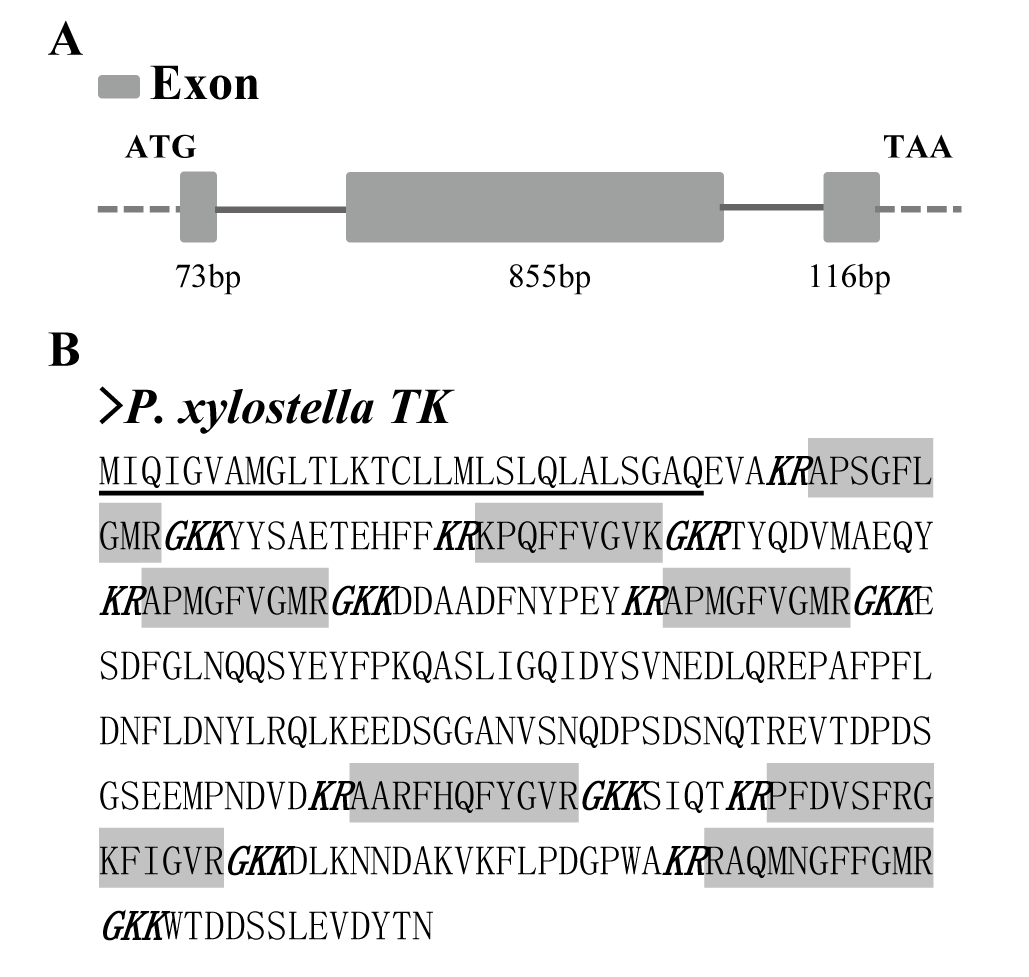

Supplement: S2 Fig — (A) The P. xylostella tachykinin (PxTK) transcript has three exons, and their nucleotide lengths are 73 bp, 855 bp and 116 bp. (B) The underlined amino acids indicate the putative signal peptide of PxTK. The gray labeled amino acids indicate the predicted mature PxTK peptides. Predicted amination amino acids (G) with dibasic cleavage sites (combinations of K and R) are in bold italics. (TIF) [file ppat.1009365.s002.tif]

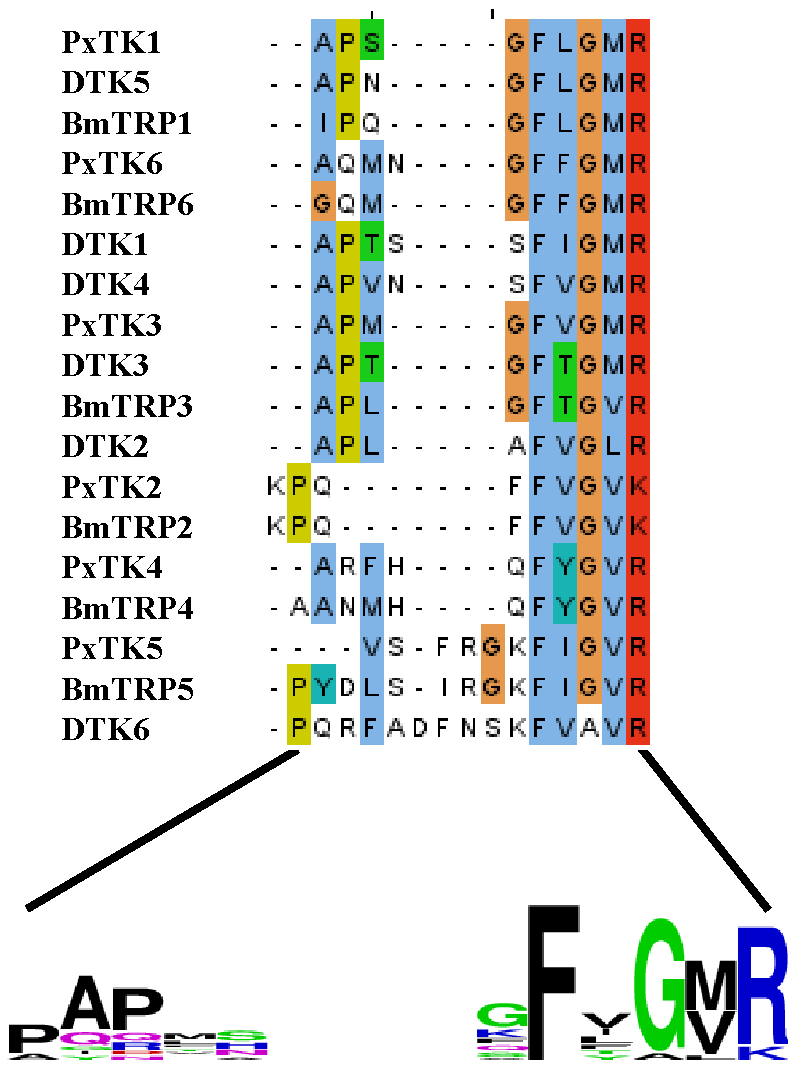

Supplement: S3 Fig — A conserved C-terminal motif is identified from each mature peptide with the sequence FxGxR (x is a variable residue). PxTK: Plutella xylostella tachykinin peptide; BmTRP: Bombyx mori tachykinin-related peptide; DTK: Drosophila melanogaster tachykinin peptide. (TIF) [file ppat.1009365.s003.tif]

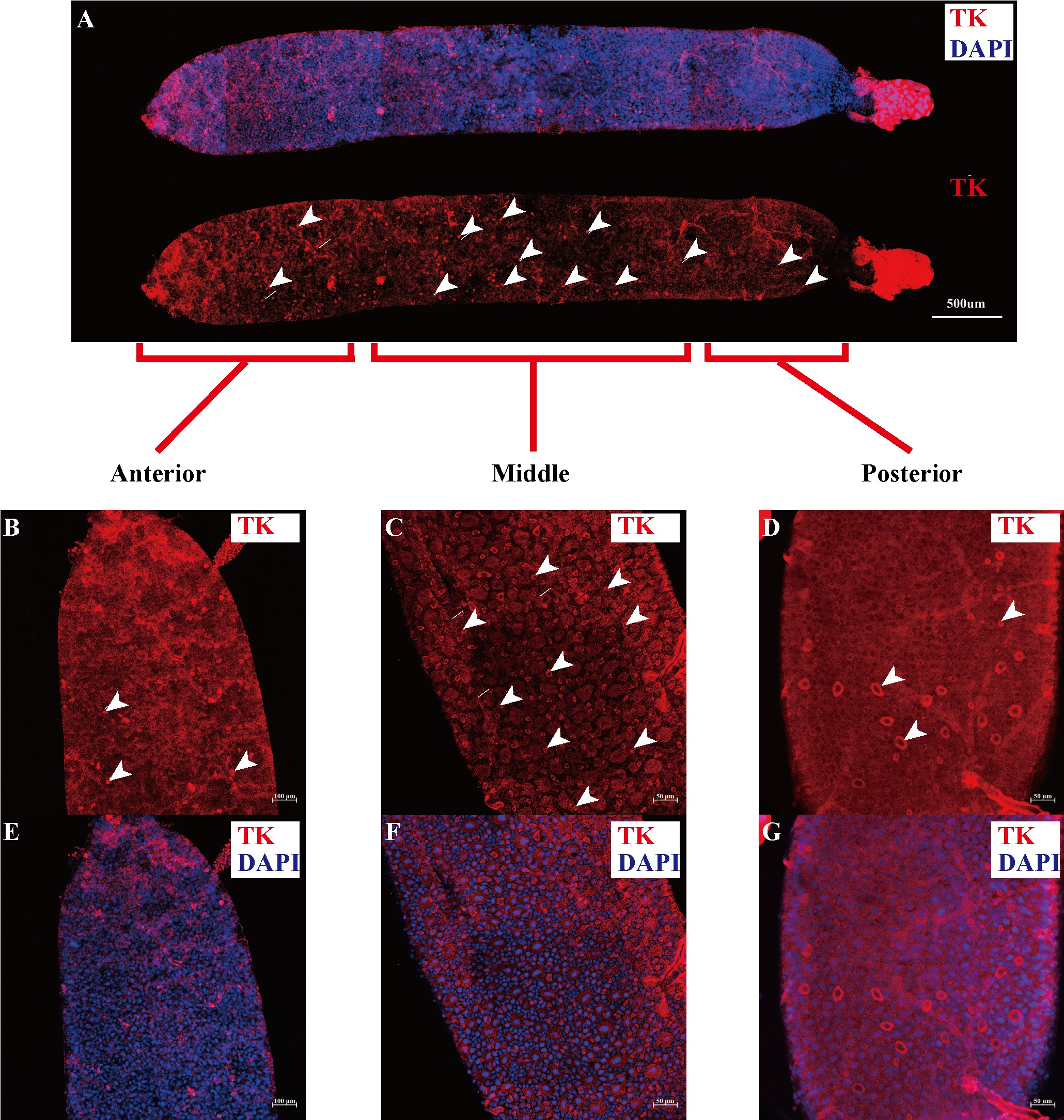

Supplement: S4 Fig — (A) Immunostaining for TK (red) in the whole midgut from 3L P. xylostella larvae. Nuclei were labeled by DAPI (blue). Scale bar: 500 μm. The number of TK-labeled cells was greater in the middle region of the P. xylostella midgut (C) than in the anterior (B) and posterior parts (D). The TK-secreting cells are indicated by arrowheads in B, C and D. Scale bars: 50 μm. 3L: Late 3rd instar. (TIF) [file ppat.1009365.s004.tif]

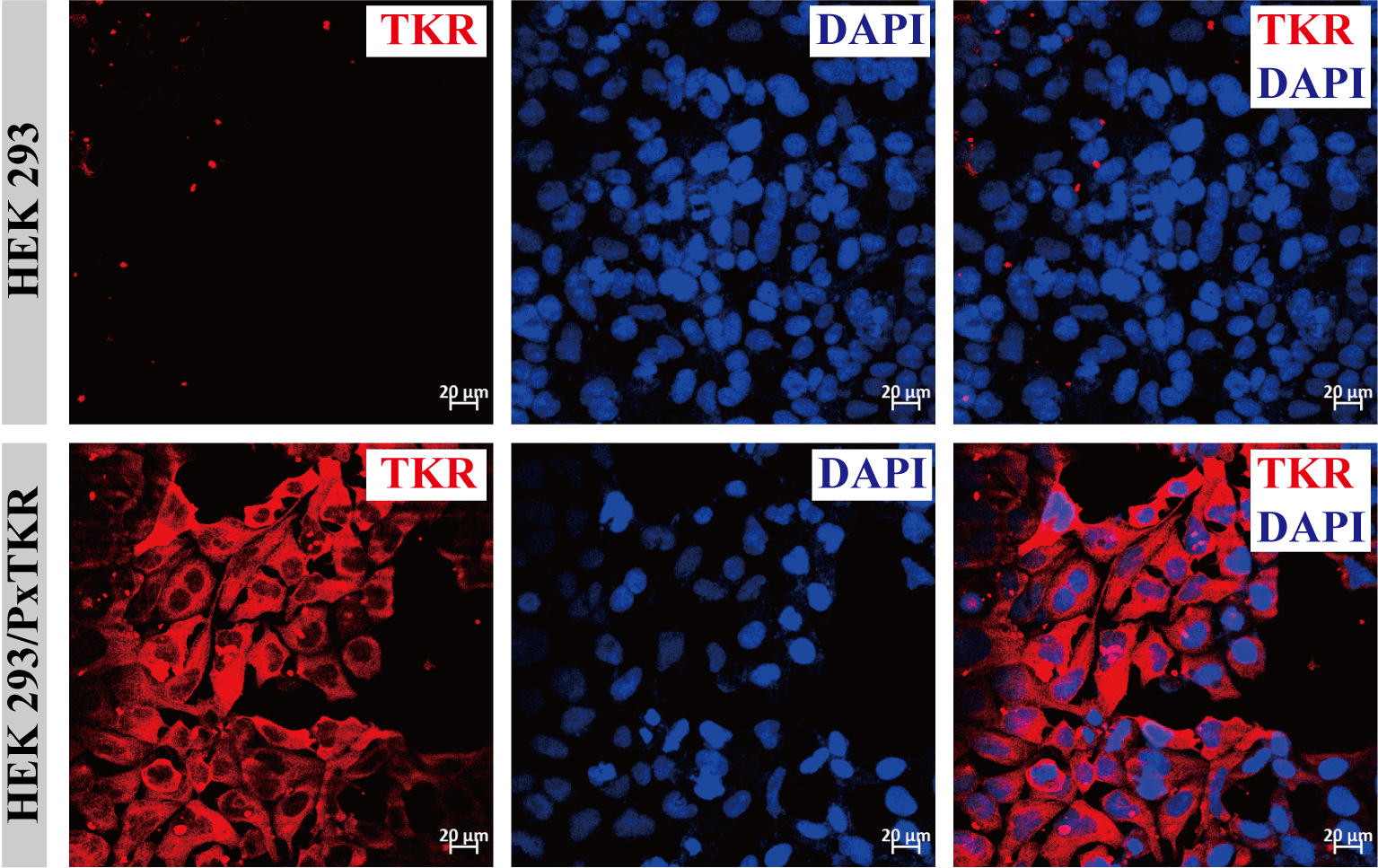

Supplement: S5 Fig — P. xylostella tachykinin receptor (PxTKR) was subcloned into the pcDNA 3.1(+) plasmid and then transfected into HEK293 cells. PxTKR was localized on the membrane in HEK293/PxTKR cells but not in HEK293 control cells (anti-TKR, red). Nuclei were labeled by DAPI (blue). Scale bars: 20 μm. (TIF) [file ppat.1009365.s005.tif]

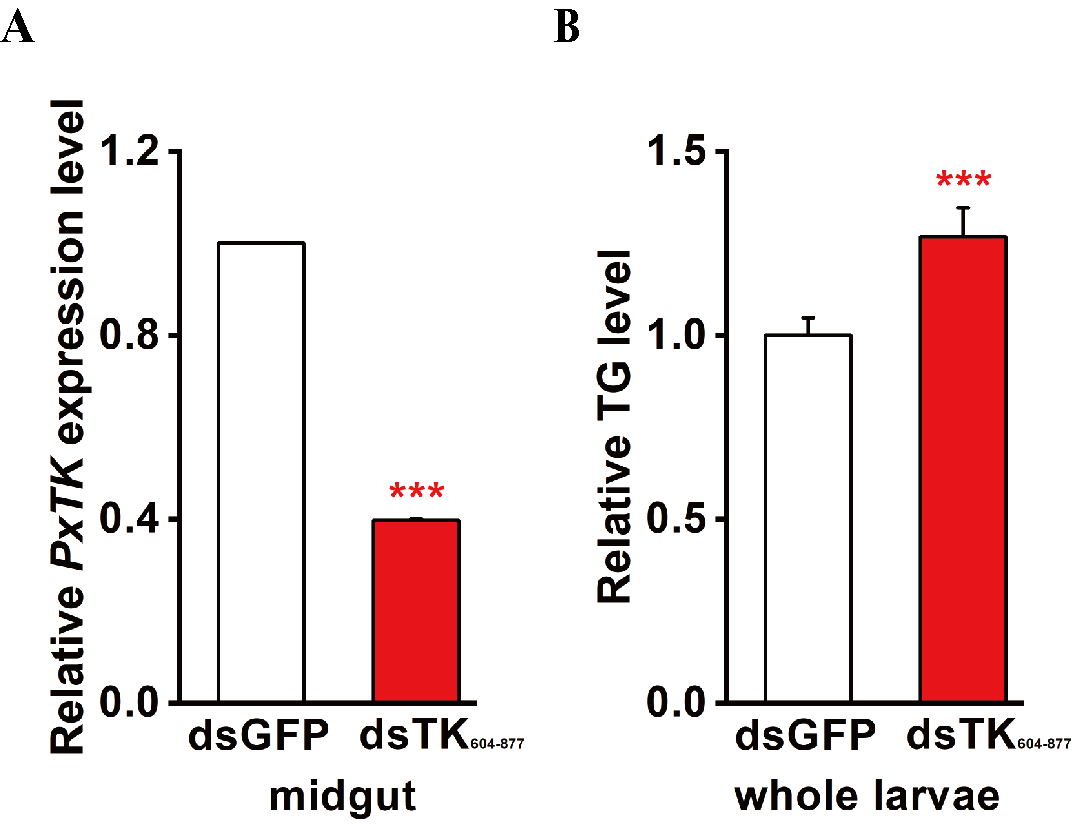

Supplement: S7 Fig — (A) Relative mRNA levels of TK in P. xylostella midgut at 3 days post PxTK604-877 dsRNA silencing with dsGFP treatment as a control (n = 5 for each group). Three biological replicates were performed. Data are the means ± SD; significance was determined by Student’s t-test (***: p < 0.001). (B) Relative levels of triglycerides (TGs) from dsPxTK604-877- and dsGFP (control)-treated P. xylostella larvae at 3 days post microinjection (n = 30 for each group). Data were analyzed by Tukey’s test. Values represent the means ± SD of five independent experiments (***: p < 0.001). (TIF) [file ppat.1009365.s007.tif]

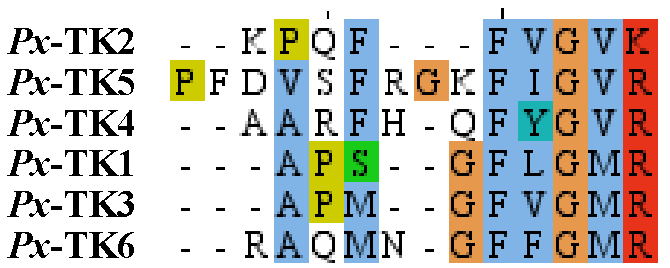

Supplement: S8 Fig — (TIF) [file ppat.1009365.s008.tif]

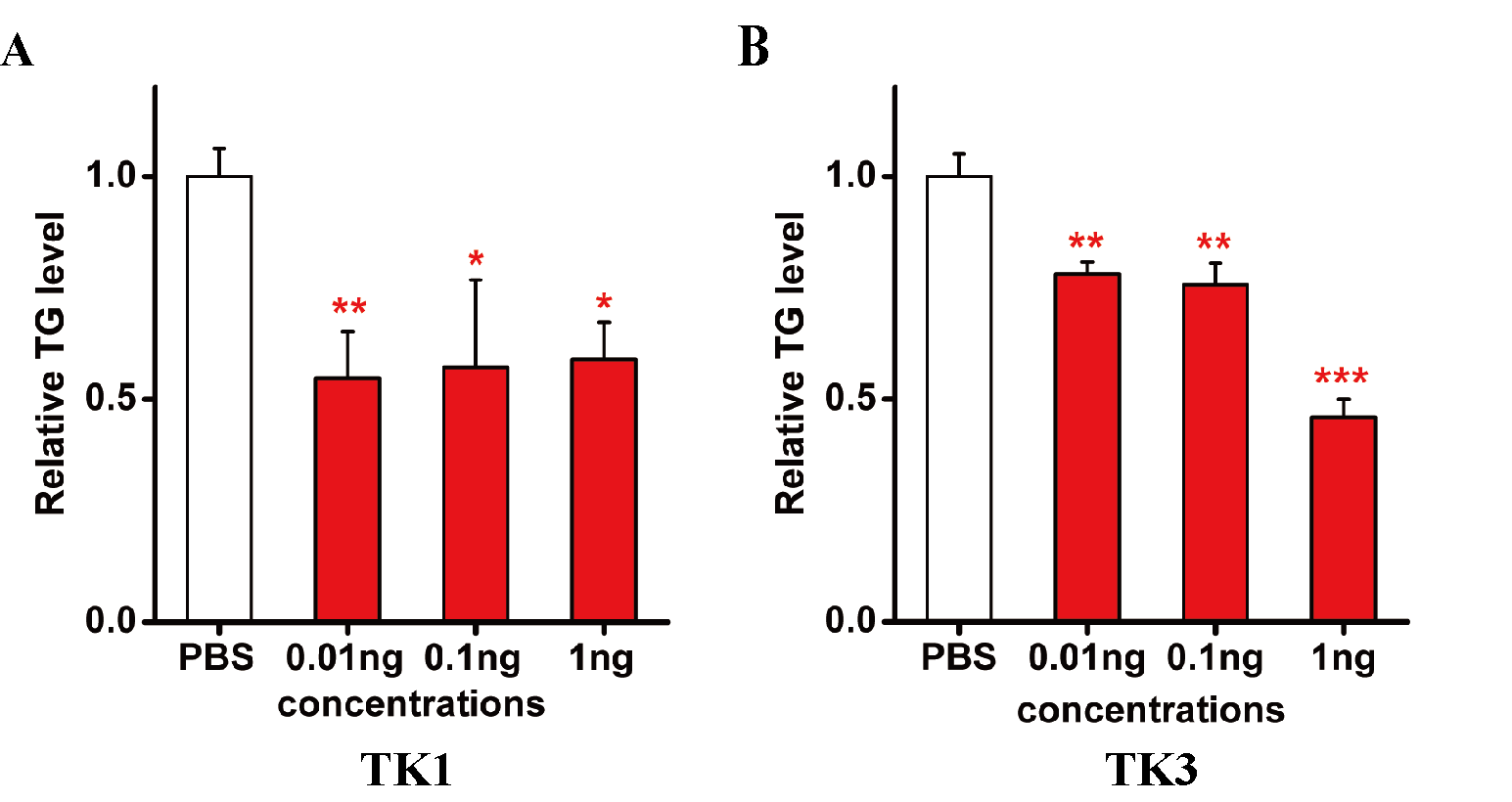

Supplement: S9 Fig — (A) Relative levels of triglycerides (TGs) from the whole body of P. xylostella larvae at 3 days post microinjection of chemically synthesized PxTK1 at different dosages (n = 5). Data were analyzed by Tukey’s test. Values represent the means ± SD of five independent experiments (*: p < 0.05; **: p < 0.01). (B) Relative levels of TG from the whole body of P. xylostella larvae at 3 days post microinjection of chemically synthesized PxTK3 at different dosages (n = 5). Data were analyzed by Tukey’s test. Values represent the means ± SD of five independent experiments (**: p < 0.01; ***: p < 0.001). (TIF) [file ppat.1009365.s009.tif]

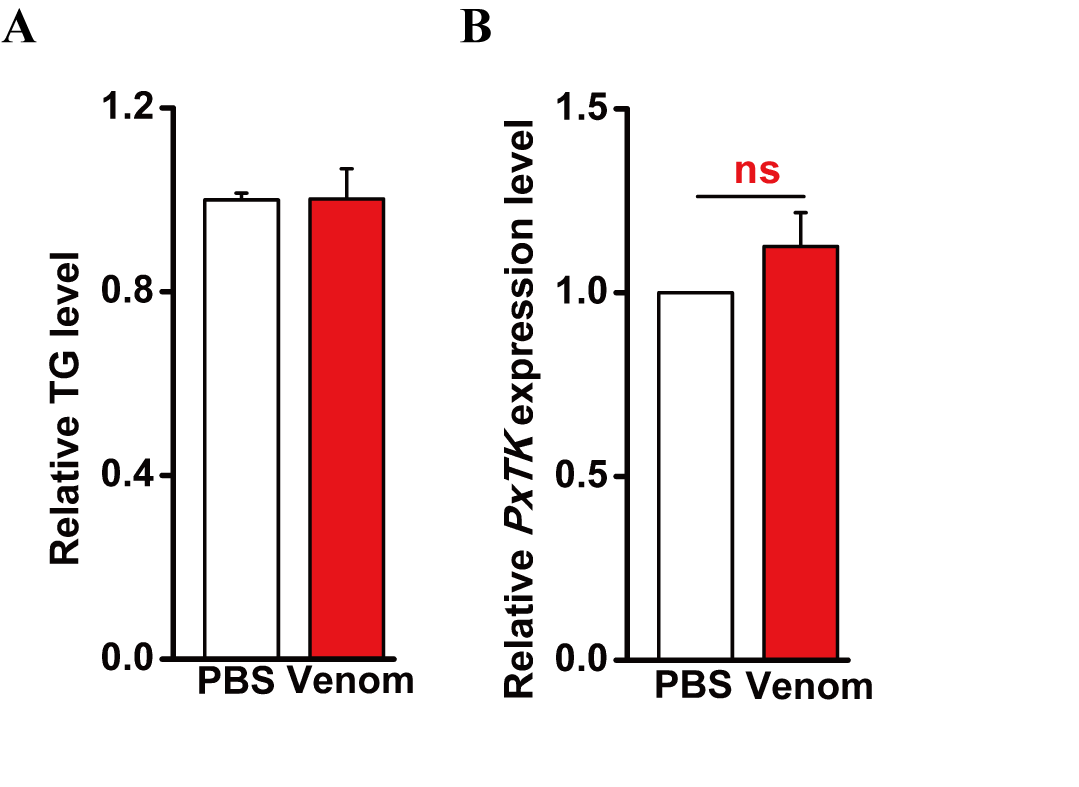

Supplement: S11 Fig — (A) Levels of triglycerides (TGs) in P. xylostella larvae at 1 day post microinjection of venom with a dose of 0.05 FE (female equivalents) (n = 10 for each group). Values represent the means ± SD of five independent experiments. (B) Relative PxTK mRNA levels in P. xylostella larvae at 1 day post microinjection of venom with a dose of 0.05 FE (female equivalents) (n = 10 for each group). Data were analyzed by Student’s t-test. Values represent the means ± SD of three independent experiments. ns: not significant. (TIF) [file ppat.1009365.s011.tif]

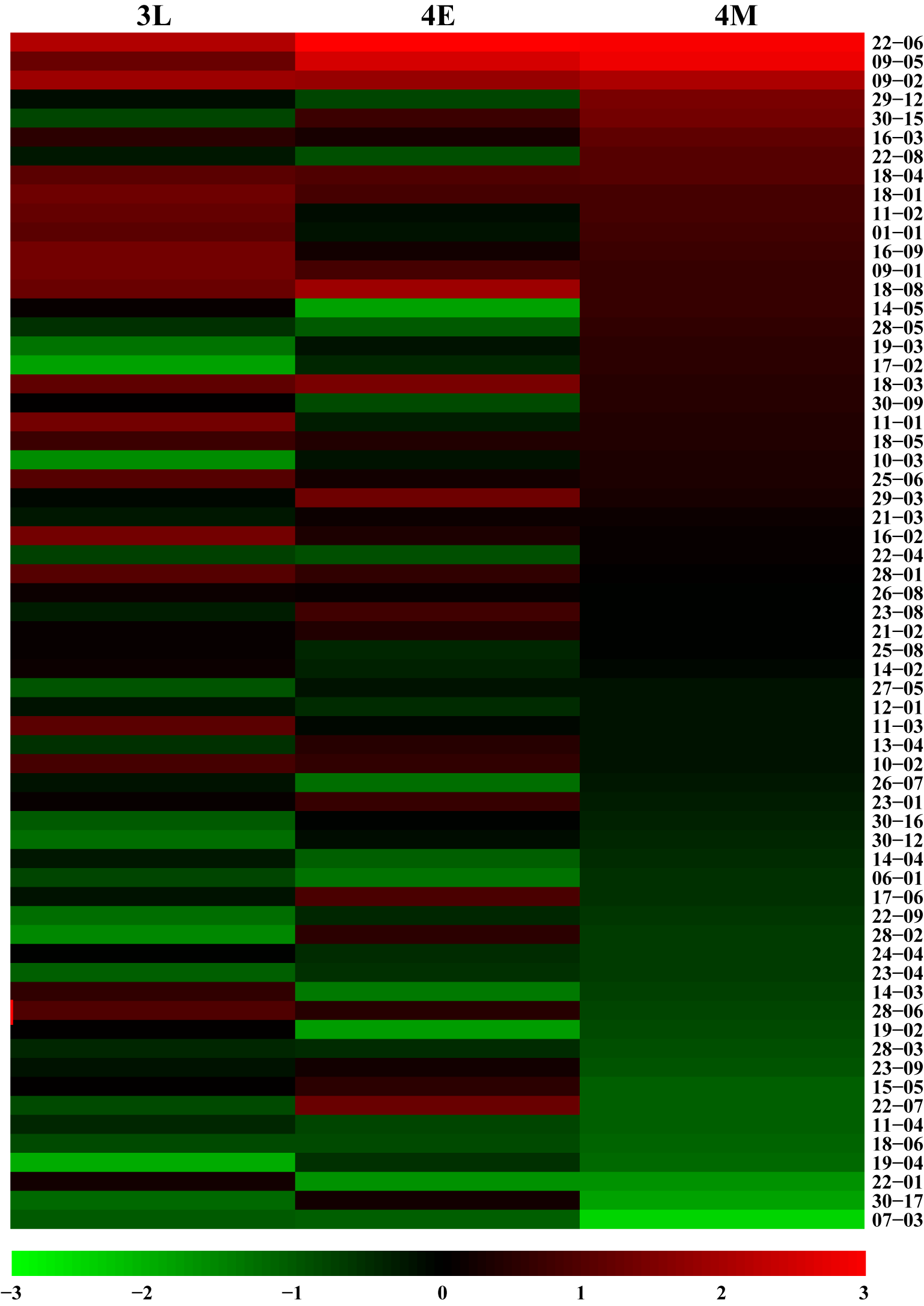

Supplement: S12 Fig — The expression profiles of C. vestalis bracovirus (CvBV) genes in the midguts of parasitized 3L, 4E and 4M host larvae. Red and green colors in the heat map indicate high and low expression levels, respectively. The most highly expressed genes in all tested development stages were CvBV 22–06, CvBV 09–05, and CvBV 09–02. The number before the short dash represents the bracovirus circle, and the number after the short dash represents the order of the genes on that circle. 3L: Late 3rd instar; 4E: Early 4th instar; 4M: Middle 4th instar. (TIF) [file ppat.1009365.s012.tif]
